# Towards the institutionalization of wastewater surveillance for public health: results from the EU-WISH mapping survey

**DOI:** 10.1093/eurpub/ckaf259

**Published:** 2026-01-14

**Authors:** Jose Antonio Baz-Lomba, Jori Perälä, Tarja Pitkänen, Tuija Leino

**Affiliations:** Department of Infection Control and Preparedness, Norwegian Institute of Public Health, Oslo, Norway; Department of Public Health, Finnish Institute for Health and Welfare, Helsinki, Finland; Department of Public Health, Finnish Institute for Health and Welfare, Kuopio, Finland; Department of Food Hygiene and Environmental Health, Faculty of Veterinary Medicine, University of Helsinki, Helsinki, Finland; Department of Public Health, Finnish Institute for Health and Welfare, Helsinki, Finland

## Abstract

Wastewater-based surveillance (WBS) is increasingly recognized as a valuable tool for monitoring public health at the population level. However, its integration into national public health frameworks across Europe remains uneven. In mid-2024, the EU-WISH Joint Action conducted a system mapping survey across 27 European countries to assess the governance, development, and integration of WBS systems. The survey combined quantitative and qualitative data to evaluate national strategies, legal and financial frameworks, and system capacities. By May 2024, most participating countries had operational WBS systems, primarily targeting SARS-CoV-2. Other monitored targets included influenza and other respiratory viruses, poliovirus, antimicrobial resistance (AMR), emerging pathogens, illicit drugs, and health-related biomarkers. Prioritization in system design was largely based on operational feasibility and perceived public health value. Challenges identified included fragmented governance, lack of sustainable financing, and limited workforce capacity. Integration into public health decision-making varied, and dissemination practices differed significantly across countries and surveillance targets. The EU-WISH survey provides a baseline assessment of WBS implementation across Europe and highlights key enablers and barriers to its institutionalization. The findings support ongoing efforts at national and EU levels to enhance coordination, sustainability, and integration of WBS into routine public health frameworks.

## Introduction

Wastewater-based surveillance (WBS) has emerged as a valuable public health tool for monitoring population-level health trends, offering anonymized insight into pathogen circulation and health-related biomarkers. Historically, WBS has been used to monitor poliovirus circulation and detect enteric pathogens [1, 2]. Its utility lies in detecting pathogens excreted in human waste, providing early warning signals independent of clinical testing or healthcare access [3].

The coronavirus disease 2019 (COVID-19) pandemic accelerated WBS implementation globally [4]. In many countries, including those in Europe, wastewater monitoring was rapidly scaled up to track severe acute respiratory syndrome coronavirus 2 (SARS-CoV-2) and emerging variants, demonstrating technical feasibility and public health value [5–7]. In the USA, the National Wastewater Surveillance System expanded from a handful of sites to over 1500 by 2022, covering nearly half the population [8]. Beyond SARS-CoV-2, WBS is now applied to monitor other infectious threats, including antimicrobial resistance (AMR) [2, 9, 10], and has long been used for tracking chemical and lifestyle indicators such as illicit drugs, alcohol, tobacco, and pharmaceutical residues [11, 12].

Recent studies highlight its relevance in settings with limited clinical infrastructure, positioning WBS as a climate-resilient and equitable approach for underserved communities and low- and middle-income countries (LMICs) [13–15]. Its potential in early detection of emerging infectious diseases and zoonotic spillovers supports its role as a One Health tool for pandemic preparedness [15, 16]. While metagenomic tools for WBS are enabling broad-spectrum detection, challenges remain in standardization and data interpretation [17, 18].

Despite growing interest and technical advances, integrating WBS into routine public health systems faces structural and financial obstacles. These include fragmented governance, inconsistent funding models, and varying institutional readiness across countries. As demonstrated during the pandemic, countries differ in their capacity to institutionalize WBS and sustain operations beyond crisis periods [19–21].

The revision of the Urban Wastewater Treatment Directive (UWWTD), particularly Article 17, introduces a legislative requirement for European Union (EU) Member States to establish WBS systems and integrate them into national public health frameworks [22]. This regulatory development presents both an opportunity and a mandate to build sustainable systems that move beyond emergency pandemic response and become part of routine public health preparedness.

To support this effort, the EU-funded Joint Action EU-WISH (EU-Wastewater Integrated Surveillance for Public Health) conducted a system mapping survey across 27 countries, aiming to assess governance, strategies, capacities, and integration of WBS. This article presents the results of that survey and identifies key challenges and opportunities for advancing WBS as a routine public health component in Europe.

## Methods

### Survey design

The system mapping survey was developed by the EU-WISH Task 5.1 working group and shared with all task leaders for review. The objective was to collect structured information on the development, governance, and integration of WBS systems across participating countries.

The questionnaire included multiple-choice and open-ended questions to capture quantitative indicators and qualitative insights. The full set of questions, grouped into four thematic areas, is available in Supplementary Table S1. These areas were aligned with EU-WISH’s key performance indicators and drew on previous surveys shared by DG HERA and the Joint Research Centre (JRC). Informed by these earlier exercises, predefined response options were used where cross-country comparability was essential, while open fields allowed respondents to elaborate or add considerations. An ‘operative WBS system’ was defined as any WBS programme implemented at research or national public health level, whether temporary or institutionalized, and generating data used for exploratory assessment or reporting to decision-makers.

### Respondents and data collection

The survey was distributed to national beneficiaries from 26 EU-WISH countries across the EU and European Economic Area (EEA): Austria, Belgium, Croatia, Cyprus, Czechia, Denmark, Estonia, Finland, France, Germany, Greece, Hungary, Ireland, Italy, Latvia, Lithuania, Luxembourg, Malta, Netherlands, Norway, Portugal, Romania, Slovenia, Spain, Sweden. Ukraine also participated in the survey as a non-EU/EEA country. Additionally, the Slovak Republic was invited by Czechia and submitted a national response, bringing the total to 27 participating countries.

Each country designated one respondent to submit a coordinated national response, typically from the public health institute. Respondents were encouraged to consult relevant stakeholders (e.g. public health, environment, wastewater operators, academia) and national workshops were recommended to facilitate data collection and expert dialogue. Ten countries organized a workshop or multi-stakeholder consultation, while others gathered inputs through email exchanges, internal discussions, or smaller meetings (Supplementary Table S2).

The survey was implemented via the EU-Survey platform, hosted by the European Commission’s Directorate-General for Digital Services (DG DIGIT), and remained open from 14 June to 31 July 2024. Due to the summer holiday period, late responses were accepted until 31 August 2024. A privacy statement was provided to all participants in line with Regulation (EU) 2018/1725. The Norwegian Institute of Public Health served as data controller. Personal data, including respondent and institutional contact details, were used only for Task 5.1, stored securely on EU-Survey servers and EU-WISH partner institutions, and accessed exclusively by authorized personnel.

### Data processing and analysis

**Figure 1.**
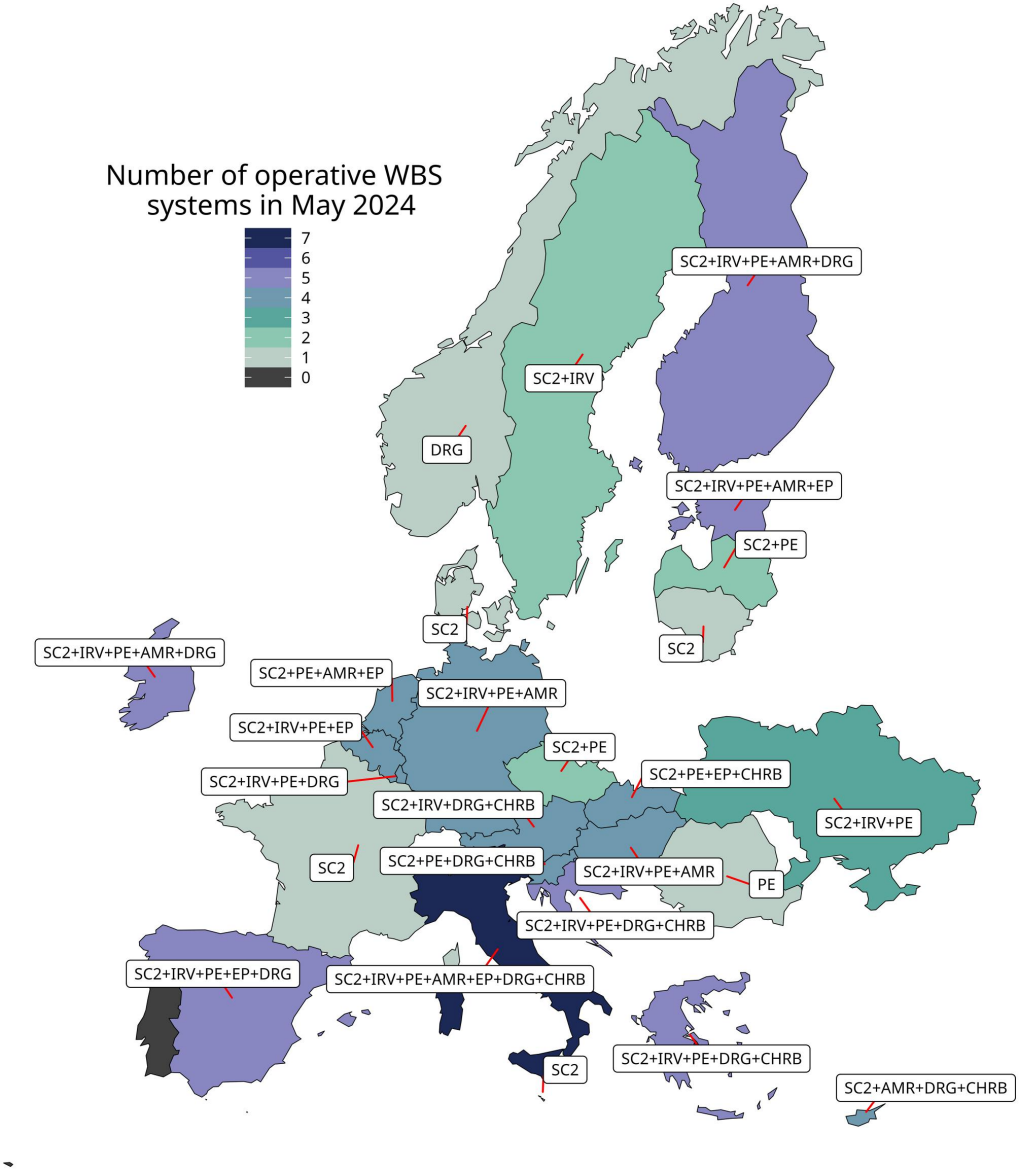
EU-WISH participating countries with operative WBS systems in May 2024 based on national responses to the EU-WISH mapping survey. Countries could report more than one monitored target. The map shows the total number of active WBS systems per country (colour gradient) and the specific targets monitored, using abbreviations for SARS-CoV-2 (SC2), influenza or other respiratory viruses (IRV), poliovirus and non-polio enteroviruses (PE), antimicrobial resistance (AMR), emerging pathogens (EP), illicit drugs (DRG), and chemicals and health-related biomarkers (CHRB).

Responses were downloaded from the EU-Survey platform and screened for completeness. Quantitative data were analysed in R (version 4.4.1) to generate descriptive statistics and visualizations. Open-ended responses were analysed thematically using a mainly deductive approach aligned with the questionnaire structure, with additional categories created when respondents raised issues not covered by the predefined themes. Qualitative data contextualized and complemented quantitative findings. To preserve confidentiality, country names and stakeholder-level information are not disclosed except in Fig. 1, and no results are attributed to individuals.

## Results

### Governance and financial aspects of sustainability

Of the 27 participating countries, 16 reported having national public health preparedness plans, of which six explicitly included WBS as a formal component. In terms of governance plans, eight countries had either developed or were developing specific national plans for WBS. A designated authority responsible for overseeing and coordinating the national WBS programme existed in 18 countries (67%).

However, several governance elements were reported as underdeveloped. Only five countries (19%) had procedures for evaluating the effectiveness and efficiency of their WBS governance structure, and only seven countries (26%) had established legal frameworks or regulations governing WBS implementation and operations. Nevertheless, most countries (81%, *n* = 22) had formal channels for collaboration, communication, and information sharing among relevant stakeholders. Mechanisms to ensure transparency and accountability in decision-making existed in 11 countries (41%), and nine countries (33%) had measures to address potential conflicts of interest among stakeholders.

Health system financing models varied. Eleven countries (41%) operated under a national health insurance model, followed by Beveridge-type systems (19%, *n* = 5), Bismarck-type systems (11%, *n* = 3), hybrid Beveridge-Bismarck models (7%, *n* = 2), and other mixed models (7%, *n* = 2). Four countries were unsure of the financing model in use. Only four countries (15%) reported having dedicated funding mechanisms for the development, maintenance, and expansion of WBS. Fourteen countries (52%) had funding mechanisms for selected WBS activities only, while seven countries lacked any established funding structure. Two countries were uncertain about their funding situation.

Cost data varied substantially by country and target area (Table 1). Among the 24 countries with active WBS for SARS-CoV-2, 13 provided budget figures, with a median estimate of around €920 000. For influenza or other respiratory viruses (RVs), 4 out of 14 countries with active systems reported a median of approximately €50 000. In the case of poliovirus and non-polio enteroviruses (NPEVs), 11 of 18 active systems provided estimates, with a median annual cost of about €40 000. Only two countries reported costs for AMR, with a median budget of roughly €110 000. One country reported an annual cost of €32 000 for emerging pathogen surveillance. For illicit drug monitoring, 6 of 11 systems shared figures, indicating a median annual budget of approximately €28 000. Lastly, two countries provided data for chemicals and health-related biomarkers (HRBs), with a median cost of around €3500.

**Table 1.** National annual budget estimates for wastewater-based surveillance systems.^a^

Target	Active systems	Number of responses	Median annual budget (EUR)	Minimum	Maximum
SARS-CoV-2	24	13	919 571	600	3 120 002
Influenza/RVs	14	4	50 000	20 000	83 878
Poliovirus/NPEVs	18	11	40 000	2455	230 000
AMR	8	2	109 500	94 000	125 000
Emerging pathogens	6	1	32 000	32 000	32 000
Illicit drugs	11	6	27 500	85	230 000
Chemicals and HRBs	7	2	3542	85	7000

a Medians reflect only the countries that reported budget figures for each target area. RVs; respiratory viruses other than influenza, NPEVs; non-polio enteroviruses, AMR; antimicrobial resistance, HRBs; health-related biomarkers.

Some countries with active systems did not report budgets, either due to the absence of dedicated funding or because WBS activities were embedded within broader projects. In many cases, the same wastewater samples were analysed for multiple targets (e.g. SARS-CoV-2 and influenza), making it difficult to disaggregate costs. Funding was often sourced from temporary grants or internal budgets. Across all target areas, only three countries reported having conducted a cost–benefit analysis, covering SARS-CoV-2, poliovirus and NPEVs, or illicit drugs.

Dedicated staffing capacity for WBS varied across countries. Eleven countries reported having mixed teams including both permanent and project-funded staff, while eight operated with project-funded teams only, and six had fully permanent teams. Two countries reported having no dedicated WBS team. Among the 25 countries with a WBS team, none had teams working exclusively on WBS. In 18 countries, teams were also responsible for other public health tasks, and in seven countries, some team members had additional responsibilities beyond WBS.

### Strategies: consolidating WBS strategies at national and international level

All 27 participating countries reported having operational WBS systems in place at some point before January 2024, not limited to 2023. Among these, 96% (*n* = 26) monitored SARS-CoV-2, making it the most targeted pathogen. Other reported targets included poliovirus and NPEVs (56%, *n* = 15), illicit drugs (48%, *n* = 13), influenza or RVs (30%, *n* = 8), chemicals and HRBs (26%, *n* = 7), AMR (22%, *n* = 6), and emerging pathogens (19%, *n* = 5). Additional targets, such as adenovirus, measles, cholera, norovirus, or monkeypox virus, were reported by five countries.

As of May 2024, 26 of the 27 countries continued to operate WBS systems (Fig. 1). However, monitoring scope had evolved compared to previous years. SARS-CoV-2 surveillance remained active in 24 countries, while two had discontinued it. Surveillance of influenza or RVs increased from 8 to 14 countries, poliovirus and NPEVs increased from 15 to 18, and AMR increased from 6 to 8. Emerging pathogen surveillance increased from five to six countries, while surveillance of illicit drugs decreased from 13 to 11 countries. WBS of chemicals and HRBs remained stable in seven countries.

**Figure 2.**
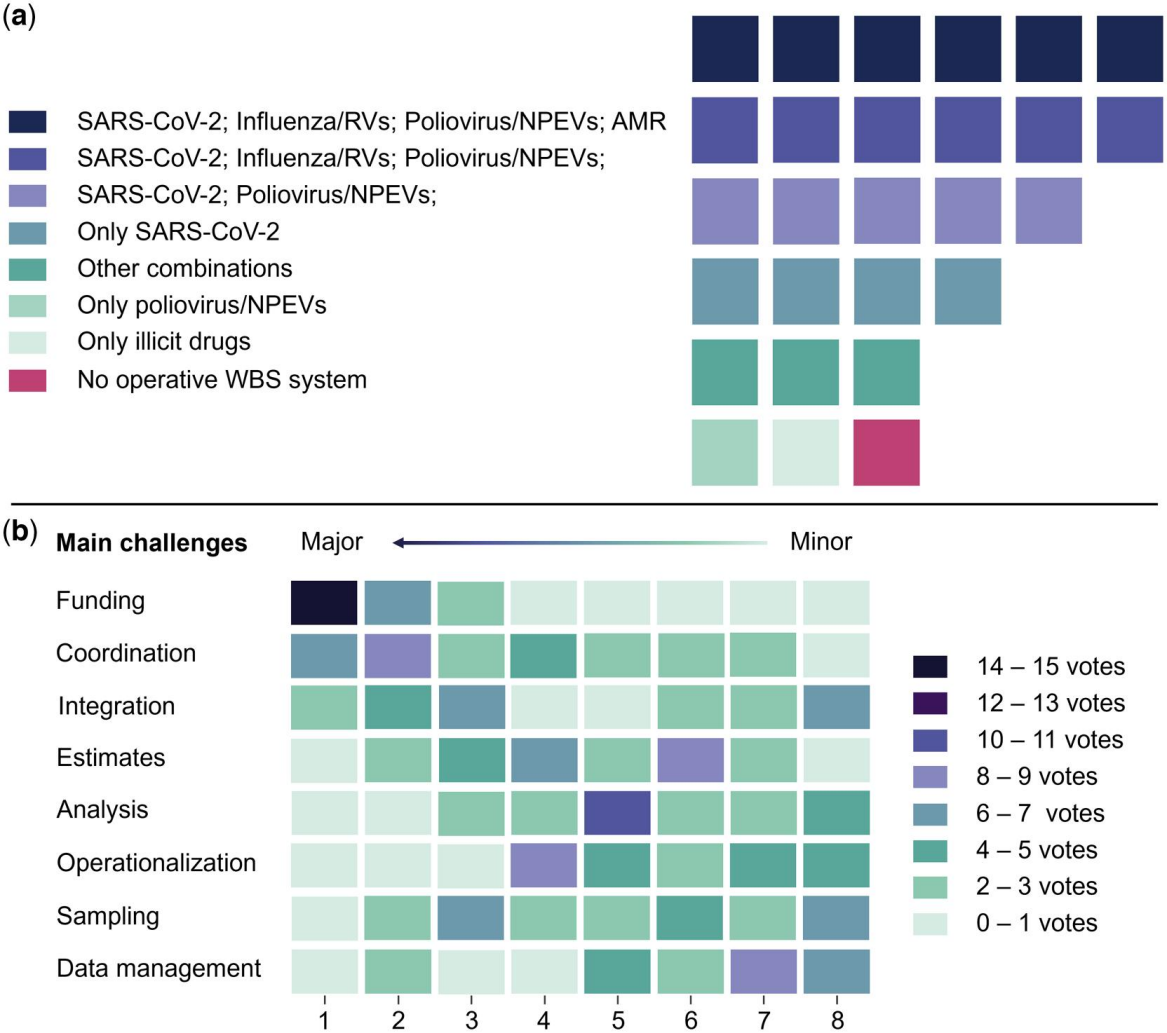
Distribution of operative WBS target combinations across countries in May 2024. Each square represents one country, grouped by similar reported combinations (a). Ranking of the main challenges reported for implementing WBS systems. Each country was asked to rank eight predefined challenges from 1 (major challenge) to 8 (minor challenge). The heatmap shows the number of countries assigning rank to each challenge, with darker colours representing higher concentrations of votes, e.g. 15 countries ranked “funding” as their major challenge to implement their WBS system (b).

The most frequent combination of surveillance targets was SARS-CoV-2, influenza or RVs, poliovirus and NPEVs and AMR, monitored concurrently in six countries, often alongside additional targets. Another six countries monitored SARS-CoV-2, influenza or RVs, and poliovirus and NPEVs. An additional five countries monitored both SARS-CoV-2 and poliovirus and NPEVs. Four countries monitored only SARS-CoV-2. Three countries followed other combinations, while the remaining two monitored only poliovirus and NPEVs or illicit drugs, respectively. One country had no active WBS system in May 2024 (Fig. 2a).

Regarding the use of WBS to detect cross-border threats at points of entry or during international events, 15 countries (56%) reported experience with such applications. However, only three had established formal guidelines for implementing measures based on positive WBS results. Six countries had integrated WBS at point of entry as a permanent feature of their surveillance system, while the remaining nine used it on an *ad hoc* basis. Reasons for activation included Public Health Emergency declarations, epidemiological assessments, international or mass events, and context-specific government decisions.

The most frequently targeted agents at point of entry were SARS-CoV-2 (*n* = 11), poliovirus and NPEVs (*n* = 6), and emerging pathogens such as measles or monkeypox virus (*n* = 5). Points of entry or high-risk settings included airports and commercial aircraft (*n* = 11), large events and international festivals (*n* = 5), and sites such as refugee camps or migrant detention centres (*n* = 5). Additionally, two countries reported WBS at land borders, and two at public transport hubs or maritime ports. Fourteen countries reported participation in the European Super Site Network sentinel system, organized under the EU Wastewater Observatory for Public Health, which aims to monitor strategic transportation hubs for pathogens and other health-related indicators.

### Strategies: plans and challenges

Most countries (*n* = 20) reported plans to expand their WBS capacities by late 2024 or early 2025. Influenza or RVs were the most frequently mentioned targets for new WBS systems (*n* = 11), followed by AMR (*n* = 9), poliovirus and NPEVs (*n* = 9), chemicals and HRBs (*n* = 8), illicit drugs (*n* = 7), and emerging pathogens (*n* = 7). Specific emerging pathogens included measles virus, norovirus, hepatitis A and E viruses, rotavirus, adenovirus, oncogenic viruses such as herpesvirus and papillomaviruses, West Nile virus, *Cryptosporidium*, avian influenza, and others. Six countries also planned to expand existing SARS-CoV-2 monitoring. Five countries were uncertain about their plans, while two reported no intentions to implement new WBS systems.

Countries were asked to rank eight predefined challenges for WBS implementation and integration. Results varied according to the level of maturity and institutionalization of the approach in each country. As shown in Fig. 2b, funding emerged as the most frequently identified top barrier (ranked first by 57%, *n* = 15), followed by coordination across institutions and sectors (26%, *n* = 7), and integration into routine public health practice (11%, *n* = 3). Less frequently cited challenges included data management, sampling and operationalization.

### Capacities: enhancing and extending national capacities

In most countries, wastewater sampling was conducted by water utilities or authorities (59%, *n* = 16), jointly by water and public health authorities (22%, *n* = 6), or by public health institutions alone (19%, *n* = 5). Sampling frequency, scale, and coverage varied considerably across countries and target pathogens (Table 2).

**Table 2.** Overview of national WBS capacities by target area (reflecting the situation in May 2024).^a^

Feature	SARS-CoV-2	Influenza/RVs	Poliovirus/NPEVs	AMR	Emerging pathogens	Illicit drugs	Chemicals and HRBs
Countries with active WBS systems in 2024	24	14	18	8	6	11	7
Sampling frequency	Weekly or more	Weekly or more	Monthly or variable	Variable	Low	Once per year	Low
Monthly samples processed	3–1844	4–320	2–250	3–92	10–124	4–200	4–40
Number of WWTPs	1–311	1–40	1–37	1–33	3–13	1–38	1–38
Approximate population covered	100 000–10 000 000	100 000–5 000 000	300 000–7 000 000	100 000–5 000 000	800 000–1 500 000	1000–10 000 000	1000–10 000 000
Number of laboratories	1–40	1–8	1–10	1–8	1–2	1–6	1
Participation in interlaboratory proficiency testing	13	2	7	0	0	10	3
Performed *in silico* ring trials on sequencing analysis	3	1	3	0	0	NA	NA
Adaptive sampling after positive detection	7	2	11	0	2	3	1
Ethical guidelines	1	0	1	0	0	4	2
Special settings (e.g. hubs)	6	4	4	3	2	0	1

a NA = not applicable.

WBS systems for SARS-CoV-2 were the most extensive, with 24 countries operating surveillance in May 2024. For influenza or RVs, poliovirus and NPEVs, AMR, and illicit drugs, WBS was more moderate in scale. Systems for emerging pathogens and chemical biomarkers were typically small-scale and exploratory. Sampling frequency was highest for SARS-CoV-2 and RVs, while monthly or variable strategies predominated for poliovirus and NPEVs, AMR, and illicit drugs. Monthly sample numbers ranged from just a few to over 1800, and the number of WWTPs covered ranged from 1 to >300. Population coverage varied widely, with SARS-CoV-2 systems covering more than 10 million people.

Most countries relied on one to two laboratories for each target area. Participation in interlaboratory ring trials was most common for SARS-CoV-2 and illicit drugs but limited for other targets. *In silico* ring trials on sequencing analysis were rarely used. Documented adaptive sampling strategies after positive detection were most frequently reported for SARS-CoV-2 and poliovirus/NPEVs. Ethical guidelines were generally lacking, though a few countries had developed them for SARS-CoV-2 or illicit drug monitoring. Additional sampling strategies in special settings such as transport hubs or care facilities were reported by some, but not widely implemented.

When asked to prioritize criteria for spatial and temporal resolution, 44% of countries (*n* = 12) cited technical feasibility and resource availability as the primary determinant. This was followed by added value for public health decision-making (37%, *n* = 10), and population coverage (19%, *n* = 5). Legal or governance factors were rarely considered primary drivers, reflecting the operational and practical focus of WBS design.

### Awareness, stakeholder engagement

Twenty-two countries reported using SARS-CoV-2 WBS results to raise public awareness, mainly via websites, dashboards, or downloadable reports. Of these, 18 provided active links to their platforms. For influenza or RVs, five countries disseminated information publicly, four of which shared links. Nine countries used WBS data for poliovirus and NPEVs awareness, though only two provided links. Similarly, nine countries shared information on illicit drugs, including eight that provided dedicated webpages. In some cases, data on illicit drugs were also made available through the European Union Drug Agency (EUDA) website. Results of AMR, and chemicals and HRBs surveillance were not shared publicly, while one country reported publishing data on emerging pathogens.

Dissemination was often aimed at multiple audiences. Responses varied across countries, with many indicating that reporting was intended for a combination of groups such as the scientific community, decision-makers, public health authorities, the general public, and internal institutional teams.

Eleven countries confirmed using secure channels for sharing sensitive information with public safety authorities. These included early warning systems, secure email, regular coordination meetings, joint task forces, integrated information platforms, cloud-based services, and direct communication with public health officers.

Twelve countries reported plans to organize learning or communication activities in 2024, such as seminars, workshops, media interviews, or conferences, to increase awareness of WBS. Six countries described active collaboration with LMICs focused on WBS, and two countries reported broader collaborations involving LMIC partners in other contexts. In addition, 12 countries had WBS training materials available to share within the EU-WISH consortium, comprising 36 distinct resources such as protocols, presentations, and guidance documents.

## Discussion

This study complements and aligns with recent international surveys documenting the rapid expansion of WBS initiatives during and after the COVID-19 pandemic [4, 6]. While WBS implementation has increased, both studies underscore the challenges of achieving long-term institutionalization and integration into public health frameworks, with variation in governance, funding, and sampling strategies across countries.

The rapid advancement of WBS calls for continued research and development to fully realize its public health potential [19, 23]. Although SARS-CoV-2 remains the most frequently monitored target, many countries have established WBS systems for influenza or RVs, poliovirus, and illicit drugs. However, the scope and scale of these efforts vary. Some countries operate comprehensive, nationally coordinated systems, while others rely on research-driven or project-based initiatives. These findings reflect broader trends noted in previous reviews: technical capacity is expanding, but integration of WBS into routine public health systems is still limited, particularly for targets beyond SARS-CoV-2 [2, 6, 20].

Most countries in Europe reported having governance structures for WBS, with two-thirds identifying designated authorities and coordination mechanisms. However, routine evaluation procedures and legal mandates remain uncommon. Stable financing is limited, with only 4 out of 27 countries reporting dedicated funding mechanisms for system development and maintenance. Reported annual budgets varied widely, from over €900 000 for SARS-CoV-2 to less than €5000 for chemical biomarker surveillance. The reuse of infrastructure and personnel across targets also complicates cost attribution. Staffing constraints were also common. While most countries had established WBS teams, none operated with fully dedicated personnel. WBS tasks were typically integrated into broader public health roles, limiting capacity for long-term development and adaptation. Funding was often sourced from time-limited grants or internal budgets, further complicating sustainability planning. These challenges mirror those seen elsewhere, such as in the USA, where WBS often relies on short-term fiscal-year budgets [8, 24, 25].

Adaptable sampling strategies have been recognized globally as essential for responsive surveillance systems [21]. Yet in this study, few countries had formal protocols for adaptive sampling, and even fewer had ethical frameworks in place. Most design decisions were driven by operational feasibility and resource constraints, rather than legal or ethical considerations. Ethical risks related to group privacy have been increasingly discussed in the literature, especially as WBS expands into new domains [26].

Sampling practices differed by target. Surveillance for SARS-CoV-2 and influenza was high-frequency and large-scale, while monitoring of other targets remained more limited. Participation in interlaboratory proficiency testing was strongest for SARS-CoV-2 and illicit drugs, but weak or absent for other areas. Harmonized protocols and shared quality assurance mechanisms have been widely recommended to improve data comparability [7, 27, 28]. While targeted sampling at high-risk sites, such as airports or nursing homes, has demonstrated clear added value [29, 30], relatively few countries reported applying such strategies regularly. Expanding such approaches could strengthen future WBS public health responses.

Challenges reported by countries were consistent: funding, intersectoral coordination, and integration into routine workflows were most frequently cited. These findings echo earlier reports showing that technical advances alone are not sufficient to embed WBS in public health systems [6, 20]. Countries also highlighted difficulties in data interpretation and sharing, pointing to a need for common frameworks to support analysis and translation into public health action [19].

Public awareness and stakeholder engagement were most established for WBS data for SARS-CoV-2, with less visibility for AMR, chemicals, or emerging pathogens. Some countries shared illicit drug data through EUDA, illustrating cross-border reporting [11]. Beyond public reporting, 12 countries planned outreach or training activities in 2024, 6 reported WBS collaboration with LMICs, and 12 countries reported access to 36 training materials in total. These efforts provide a valuable base for scaling up capacity building and structured knowledge exchange across Europe. Stakeholder contact information collected through the survey is maintained within the EU-WISH consortium to support project communication and cannot be publicly disclosed due to confidentiality requirements. However, aggregated insights derived from these data will be reflected in future EU-WISH outputs.

The strengths of this study include broad country participation, a structured and validated survey instrument, and triangulated analysis of both quantitative and qualitative data. However, limitations include the self-reported nature of the responses, potential variability in national coordination when completing the survey, and differences in interpretation of specific survey items.

As European countries prepare to implement Article 17 of the revised UWWTD, this survey highlights both progress and persistent gaps in WBS system development. While technical capacity and national coordination have improved since the pandemic, many systems remain fragmented, target-specific, and reliant on short-term funding.

Key challenges include the absence of legal frameworks, unstable financing, and limited dedicated workforce capacity. Nonetheless, positive developments, such as increased awareness, emerging training resources, and collaboration with LMICs, offer a foundation for scaling up efforts.

To transition from emergency-driven initiatives to sustainable, integrated systems, countries must align governance, financing, and data use with public health objectives. The survey findings offer a baseline for strategic planning, shared learning, and investment prioritization. At the EU level, coordinated action and harmonized methodologies will be essential to maximize the long-term value of WBS as a pillar of public health preparedness and response.

## Supplementary Material

ckaf259_Supplementary_Data

## Data Availability

The overview data of this study is presented in the article text, figures and tables herein. The data represents the situation in the way country representatives responded during the survey. The full survey is available as Supplementary Information. Due to the survey privacy statement, the country-specific responses are not available. It is important to note that the current wastewater surveillance activity in the countries might vary from the situation in spring 2024. Key points The EU-WISH system mapping survey across 27 countries revealed that the institutionalization of wastewater-based surveillance (WBS) for public health remains limited and highly variable across Europe. Key gaps were identified in national strategies, governance structures, and sustainable financing mechanisms to support long-term WBS implementation. Strengthening WBS capacities and integrating surveillance data into public health systems is essential to support the revised Urban Wastewater Treatment Directive and broader preparedness efforts.
