# Supplementary material for: Towards the institutionalization of wastewater surveillance for public health: results from the EU-WISH mapping survey
Source: Eur J Public Health. 2026 Jan 14;36(2):ckaf259. doi: 10.1093/eurpub/ckaf259 (PMC13017784; doi:10.1093/eurpub/ckaf259)
Supplement: ckaf259_Supplementary_Data [file ckaf259_supplementary_data.zip › ejph-2025-07-om-0562-File006.docx]

**Table S1**. Overview of the sections of the survey included in this study. The numbers refer to the questions of the survey included in the Appendix. Questions referenced in this article were grouped into four thematic areas: (i) governance and sustainability: focusing on legal frameworks, coordination structures, budgeting, personnel, and financing mechanisms; (ii) national strategies and targets: including monitored pathogens and substances, surveillance objectives, and strategic approaches; (iii) capacities: such as responsible institutions for sampling, sampling frequency and number of sites, targets analysed, number of laboratories and special applications (e.g. points of entry, events); (iv) awareness and stakeholder engagement: addressing communication strategies, reporting practices, dissemination channels and training materials.

| **Governance and sustainability** | |
| --- | --- |
| 4.01 – 4.04  4.05 – 4.08  4.09 – 4.17  4.22 – 4.25  4.28  4.33 – 4.34 | Existence of national preparedness plans and inclusion of WBS  Governance: designated authority and evaluation of the WBS’ effectiveness  Legal framework  Funding mechanism  Annual budget per pathogen and existence of cost–benefit analysis  Type of WBS team and whether it is fully dedicated |
| **WBS strategies** | |
| 5.1.01 – 5.1.04  5.1.05 – 5.1.07  5.1.08 – 5.1.09  5.1.10  5.1.11 – 5.1.19 | Previous WBS systems before 2024  Active WBS system in May 2024  Plans to implement new systems in the near future  Main implementation challenges  WBS strategies to address cross-border health threats |
| **Capacities at national level** | |
| 5.2.01 – 5.2.02  5.2.03 – 5.2.04  5.2.05  5.2.06  5.2.09  5.2.10  5.2.11 – 5.2.12 | Sample collection responsibility  Sampling frequency and number of samples  WBS adaptable sampling strategies after positive detection or emergency  Laboratory capacity and QA/QC  WWTP coverage and population served  Aspects considered for prioritising spatial and temporal resolution  Additional sampling strategies and ethical guidelines |
| **Awareness and engagement** | |
| 5.3.01 – 5.3.05  5.3.06 – 5.3.12 | Use of WBS for public awareness  Training, capacity building and collaboration with LMICs |
