# Supplementary material for: Towards the institutionalization of wastewater surveillance for public health: results from the EU-WISH mapping survey
Source: Eur J Public Health. 2026 Jan 14;36(2):ckaf259. doi: 10.1093/eurpub/ckaf259 (PMC13017784; doi:10.1093/eurpub/ckaf259)
Supplement: ckaf259_Supplementary_Data [file ckaf259_supplementary_data.zip › ejph-2025-07-om-0562-File008.docx]

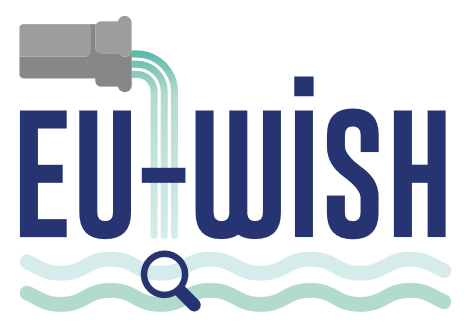


EU-WISH T5.1 Survey

Fields marked with * are mandatory.

# Welcome to the EU-WISH Survey

## Survey structure


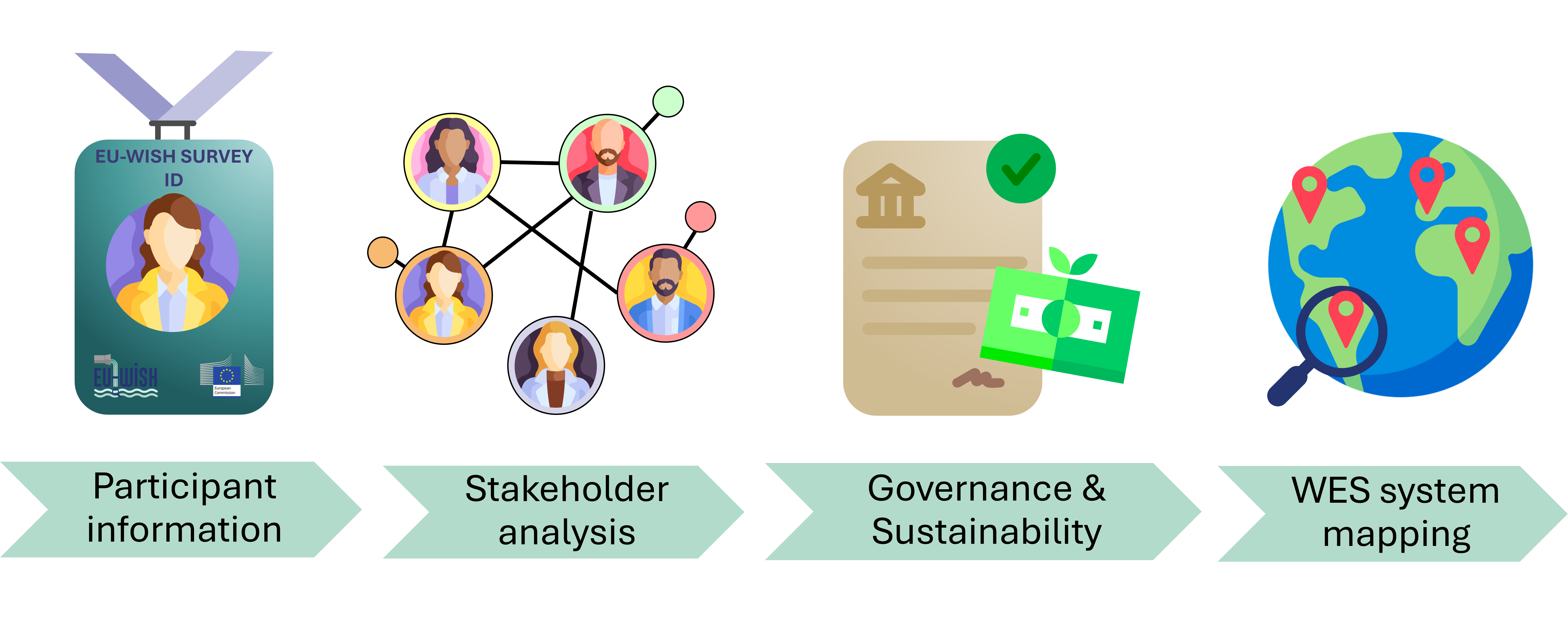


**Abbreviations**

**AMR** Antimicrobial resistance

**CCHF** Crimean Congo Hemorrhagic Fever Virus **CDC** Centers for Disease Control and Prevention **CPE** Carbapenemase-producing Enterobacterales **ESBL** Extended-spectrum beta-lactamases

**GPEI** Global Polio Eradication Initiative

**KPI** Key Performance Indicator

**RVs** Respiratory viruses

**LMIC** Low- and middle-income country

**NPEV** Non-polio enterovirus

**PCR** Polymerase chain reaction

**PEF** Poliovirus-essential facility

**PV** Poliovirus

**RCC** European Regional Commission for the Certification of Poliomyelitis Eradication

**RVF** Rift Valley fever

**VRE** Vancomycin-resistant enterococci

**WES** Wastewater and environmental surveillance

**WGS** Whole-genome sequencing **WHO** World Health Organization **WWTP** Wastewater treatment plant

## Definitions

**WES**

Wastewater and Environmental Surveillance

**Operative WES system**

Wastewater and environmental surveillance program implemented 1) either at research level or national public health level, 2) with either limited duration or institutionalized, 3) where data is either in exploratory phase or reported to key decision-makers.

**Emerging pathogens**

An emerging (or re-emerging) pathogen of human health concern in wastewater surveillance can be defined as a pathogen that, at the moment of assessment, displays at least one of the following characteristics:

1. Newly appeared in a population, including pathogens of zoonotic origin.
2. Known to occur in a population but rapidly increasing in circulation (e.g. measured as increased incidence or shedding in wastewater).
3. Known to occur in an area but expanding in geographic range.
4. Known to have occurred in a population and area in the past, declined or eradicated, but now reappearing or increasing in circulation or geographic range.
5. Having undergone genetic mutations leading to a possible increase in virulence, transmissibility, or decreased efficacy of available public health control measures. 6. Declared, or under consideration for declaration, as a Public Health Emergency by national or international health organizations.

Furthermore, to be considered as an emerging pathogen of concern for environmental surveillance under Art. 17 of the UWWD [reference to OJEU to be included], a pathogen should preferably be known or reasonably expected to be shed into wastewater through various body fluids. In addition, an emerging pathogen of concern for environmental surveillance in the context of Art. 17 of the UWWD should display features that allow environmental surveillance to provide additional, complementary or supporting information to clinical surveillance, e.g. detection of asymptomatic or subclinical infections, detection at pre- symptomatic stages, etc.

In the context of the EU-WISH project, emerging pathogens falling within the definition and already covered by tasks/sub-tasks other than 5.6 and 7.2.4 of the project (e.g. SARS-CoV-2 or multidrug resistant pathogenic bacteria) are excluded from the activities.

## Privacy statement

In giving your consent to participate in this survey, you understand that personal information collected about you, such as your name, will not be shared beyond the study team over the duration of the assignment and beyond. You understand that the information you provide will be used in reports and other deliverables of EU-WISH to aid reaching EU-WISH objectives towards integration of wastewater-based surveillance activities for public health action.

Please read below the full privacy statement providing you information about the processing and the protection of your personal data:

1.1 Privacy statement:

[Privacy_statement_EU-WISH_task_5.1.pdf](https://ec.europa.eu/eusurvey/files/78cf212e-7aea-4802-888b-114529e1006c/3ff0d81e-6e19-40dc-af9b-abce4df1819b)


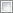
 I consent to participating in this survey according to the terms described above.

# Participant general information


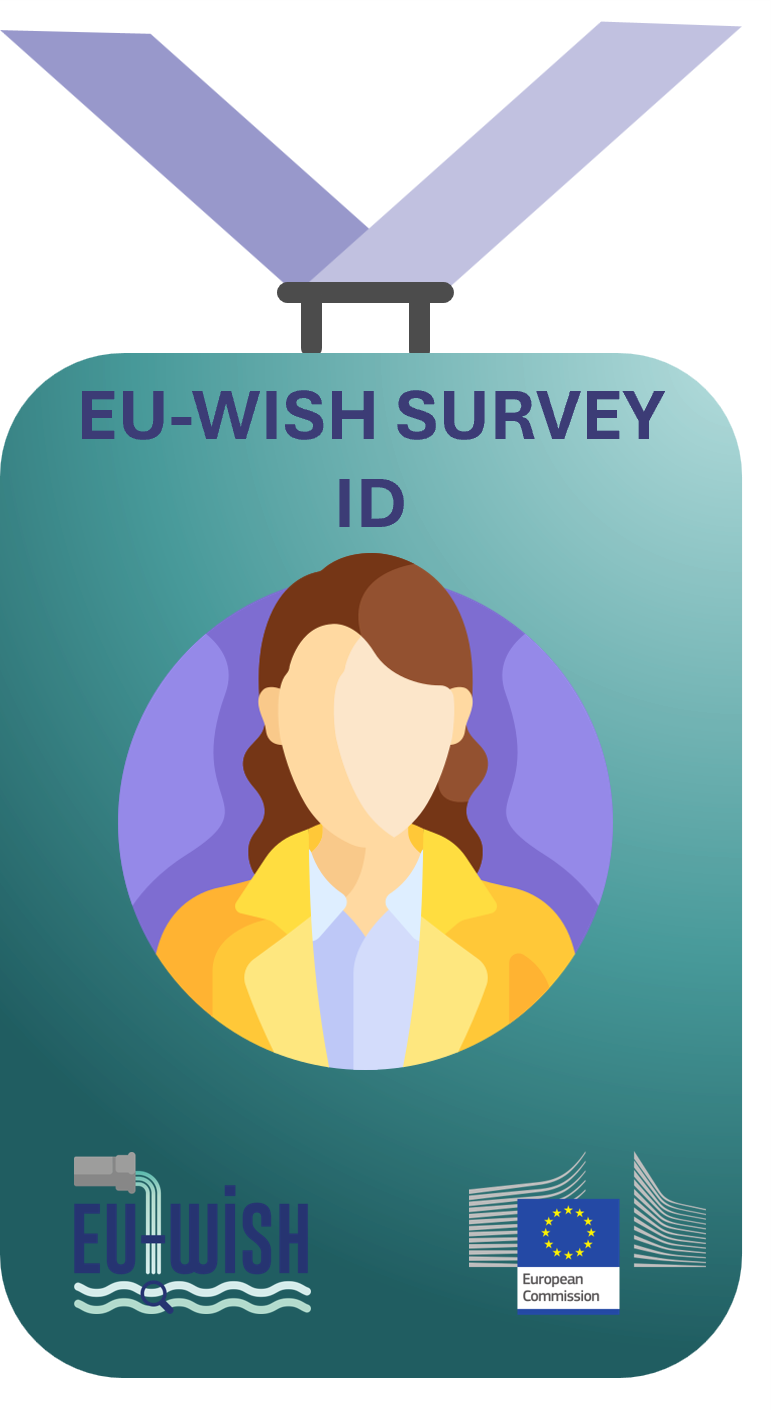


- 2.1 **Name and surname**
- 2.2 **Country**


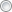
 AT - Austria
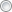
 FI - Finland
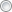
 LV - Latvia
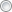
 RO - Romania


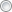
 BE - Belgium
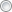
 FR - France
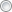
 LT - Lithuania
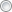
 SK - Slovak Republic
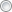
 BG - Bulgaria
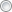
 DE - Germany
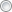
 LU - Luxembourg
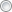
 SI - Slovenia


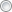
 HR - Croatia
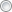
 EL - Greece
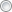
 MT - Malta
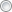
 ES - Spain


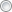
 CY - Cyprus
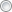
 HU - Hungary
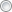
 NL - Netherlands
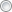
 SE - Sweden
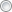
 CZ - Czechia
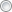
 IS - Iceland
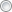
 NO - Norway
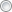
 UA - Ukraine
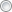
 DK - Denmark
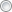
 IE - Ireland
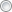
 PL - Poland


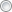
 EE - Estonia
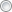
 IT - Italy
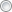
 PT - Portugal

- 2.3 **Email address**
- 2.4 **Name of institution**
- 2.5 **Type of institution**
- Ministry of Health
- Ministry of Environment


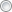
 National Public Health Institution
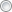
 National Reference Laboratory


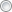
 Regional/Local Public Health Authority
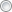
 Research Institution


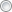
 University
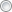
 Other

2.6 Other institution

# National stakeholder landscape


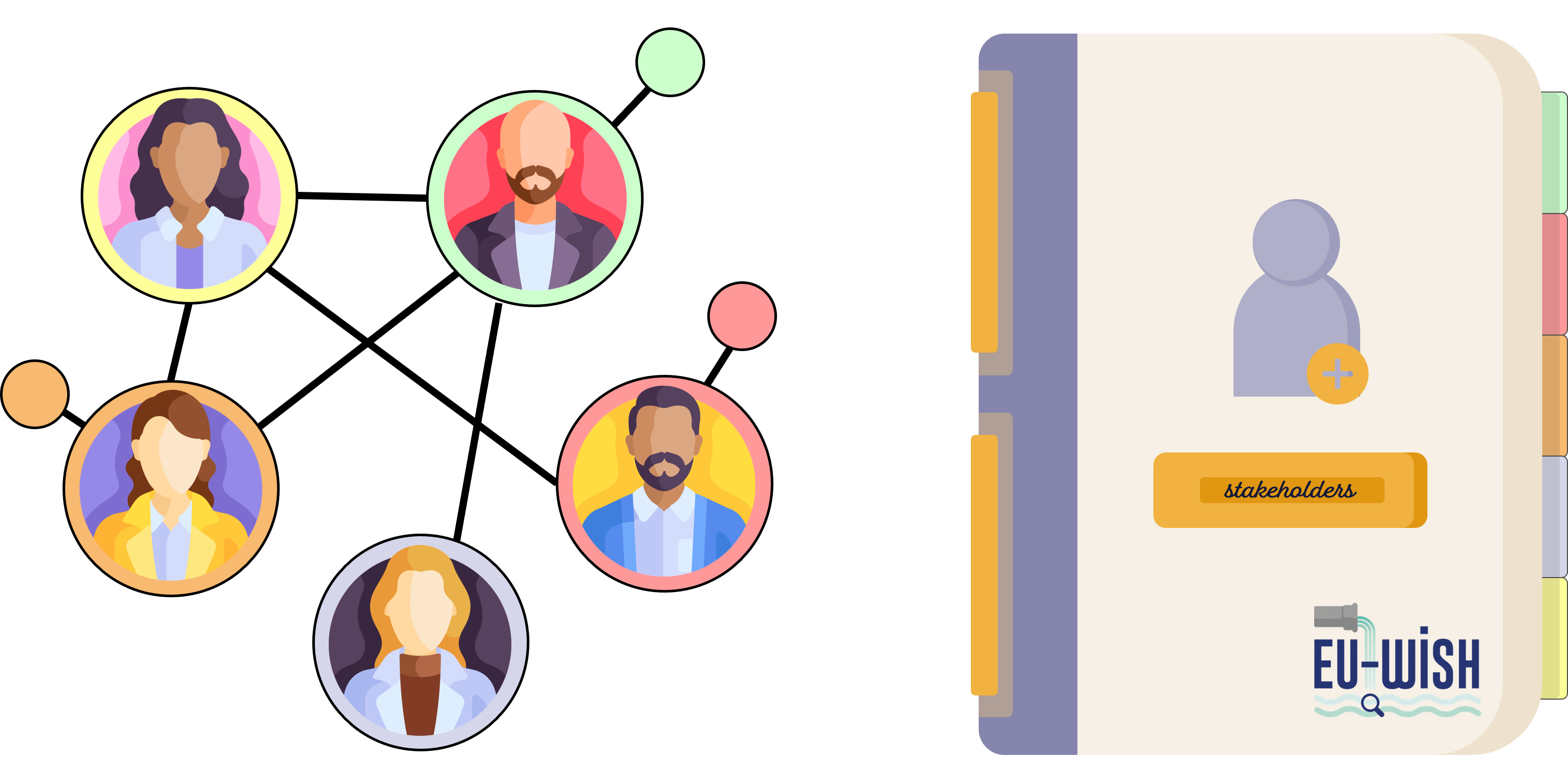


***Main objective: build a database of organizations and national contact points for an optimal and ad- hoc communication and dissemination.***

- 1. **Download excel spreadsheet**

[EU-WISH_Survey_3.2_Stakeholder_database_XX.xlsx](https://ec.europa.eu/eusurvey/files/78cf212e-7aea-4802-888b-114529e1006c/1c657ca0-fd56-4d70-95af-fe021293d127)

- 1. **Save and upload the list adding your two-letter country codes (ISO 3166-1) at the end of the file name**

**Example: *EU-WISH Survey 3.2 Stakeholder database_NO***

- 1. **Participation in other EU Initiatives - Potential synergies**

**Keep blank if not participating**

|  | Competent Authority in your country (name institution) | Contact person | Email |
| --- | --- | --- | --- |
| EU-HIP |  |  |  |
| DURABLE |  |  |  |
| WGS / RT-PCR |  |  |  |
| JAMRAI2 |  |  |  |
| United4Surveillance |  |  |  |
| One Health Surveillance  (CP-g-22-04.01) |  |  |  |

#### 6

- 1. **List of ongoing WES research projects in your country using both** [**CORDIS**](https://cordis.europa.eu/search) **and national research databases**

[EU-WISH_T5.1_Research_project_2codecountry.xlsx](https://ec.europa.eu/eusurvey/files/78cf212e-7aea-4802-888b-114529e1006c/8e0191ef-96d3-4758-9d57-48fa19a3efc5)

- 1. **Upload your list of ongoing WES research projects adding your two-letter country codes (ISO 3166-1) at the end of the file name.**

**Example: EU-WISH_T5.1_Research_project_NO**

# Governance and sustainability


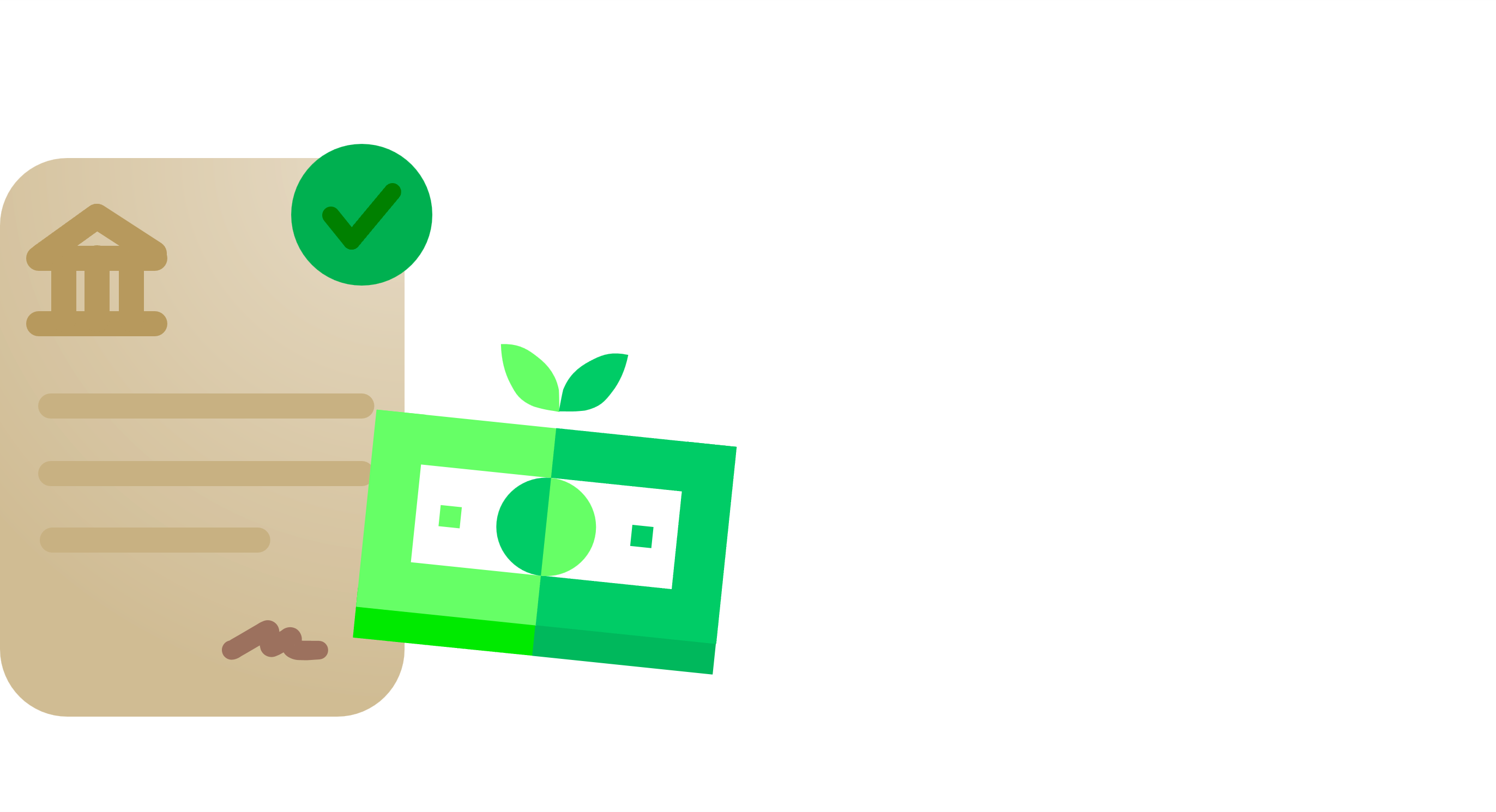


*GOVERNANCE*

- 4.1 **Do you have a National Public Health Preparedness Plan in your country?**

ECDC is currently assessing national preparedness plans. Ingrid Keller (DGSante) suggested to avoid duplication. I think it is ok to keep this questions in a simple manner.


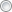
 Yes


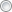
 No


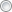
 I don't know

- 4.2 **If so, is WES part of your National Public Health Preparedness Plan?**


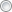
 Yes
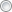
 No


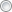
 I don't know

- 4.3 **Has a governance plan been developed for the existing or yet to be developed WES program?**


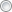
 Yes
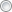
 No


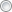
 I don't know

- 4.4 **Open field for details about the governance plans.**
- 4.5 **Is there a designated authority responsible for overseeing and coordinating governance for the national WES program within your country?**


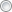
 Yes
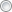
 No


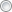
 I don't know

- 4.6 **Open field for details about designated authorities.**

**Please name them**

- 4.7 **Are procedures in place for evaluating the effectiveness and efficiency of the governance structure of your national WES program?**


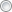
 Yes
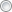
 No


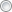
 I don't know

- 4.8 **Open field for details about the evaluation procedures.**
- 4.9 **Is there a legal framework or regulations governing the operation and implementation of your national WES program?**


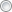
 Yes
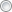
 No


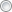
 I don't know

- 4.10 **Open field for details about legal frameworks.**

**If yes, please include the legal act with the link**

- 4.11 **Do you have established channels for collaboration, communication and information sharing among relevant stakeholders involved in WES efforts in your country?**


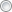
 Yes
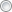
 No


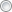
 I don't know

- 4.12 **Open field for details about channels established with stakeholder.**
- 4.13 **Are mechanisms in place to ensure transparency and accountability in the decision-making processes related to the operation and management of the national WES program?**


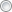
 Yes
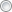
 No


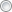
 I don't know

- 4.14 **Open field for details about those mechanisms.**

4.15 What mechanisms are in place to allow collaboration between water utilities and health utilities?

- 4.16 **Are measures taken to address any potential conflicts of interest among stakeholders involved in the governance and implementation of the WES program?**


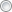
 Yes
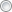
 No


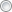
 I don't know

- 4.17 **Open field for details about measures.**
- 4.18 **Is your country participating in any other WES initiatives/programs with international partners such as WHO, European Commission or the European Drug Agency?**

**e.g. training activities, research projects, partnerships etc.**


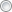
 Yes
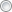
 No


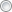
 I don't know

- 4.19 **Open field for details about other WES initiatives and international partners.**
- 4.20 **Do you engage with countries which are not part of the EU-WISH consortium and do you have mechanisms in place for this?**


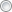
 Yes
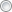
 No


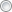
 I don't know

- 4.21 **Open field for details about engaging mechanisms.**

*SUSTAINABILITY*

- 4.22 **What is your National Health financing model?**


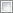
 Beveridge
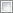
 Bismarck


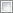
 National Health Insurance Model
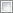
 Out-of-Pocket Model


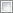
 I don't know
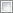
 Other

4.23 If other, please explain the model.

- 4.24 **Is there a funding mechanism or funds allocated for the development, maintenance and expansion of the wastewater surveillance infrastructure and activities?**


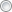
 Yes, funding mechanism established


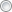
 Yes, funding mechanism establish only for some activities
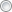
 No


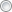
 I don't know

- 1. **Please provide more information if needed.**
  2. **Name and email of contact persons responsible for the management of the funding mechanism related to 4.24.**

**e.g. Quincy Carney;** [**Quincy.Carney@aa.com**](mailto:Quincy.Carney@aa.com)

- 1. **Is there national entity that calculates the direct and indirect costs to manage and control the different WES programs.**

**Add the names of the entities or respond "No"**

- 1. **Current overall yearly budget for the WES surveillance system and cost-benefit analysis.**

|  | Annual budget | Cost-benefit analysis performed for this system? | Open field to provide more info  i.e., aggregated estimates - cost breakdown ((lab material, human resources, operating...) |
| --- | --- | --- | --- |
| SARS-CoV-2 | Euros | 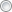 Yes 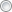 No |  |
| Influenza or other RVs | Euros | 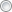 Yes 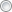 No |  |
| Polio and NPEV | Euros | 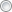 Yes 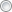 No |  |
| AMR | Euros | 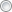 Yes 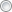 No |  |
| Illicit drugs | Euros | 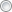 Yes 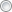 No |  |
| Emerging pathogens | Euros | 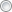 Yes 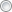 No |  |
| Chemicals and health- related biomarkers | Euros | 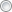 Yes 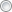 No |  |

#### 11

- 4.29 **Rank which target benefit public health authorities the most from an early detection in wastewater?**

*Use drag&drop or the up/down buttons to change the order or accept the initial order.*


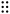


**Chemicals and health-related biomarkers**

**Emerging pathogens**

**Illicit Drugs**

**AMR**

**Polio and NPEV**

**Influenza or other RVs**

**SARS-CoV-2**

4.30 Please provide the rational for the rank above.

- 4.31 **Sort targets by their potential impact in the national health budget based on the overall complexity of the WES approach (e.g. sampling, analysis, data modelling).**

From the ones the result in higher costs (top) to the ones with less impact in the health budget (bottom).

*Use drag&drop or the up/down buttons to change the order or accept the initial order.*

**Chemicals and health-related biomarkers**

**Emerging pathogens**

**Illicit Drugs**

**AMR**

**Polio and NPEV**

**Influenza or other RVs**

**SARS-CoV-2**

4.32 Please justify the rank above i.e., main factors impacting the cost of your surveillance

- 4.33 **Is there an assigned personnel and/or dedicated team of experts for conducting WES? This can be at the national or subnational level.**

Yes, a permanent team

Yes, a project-financed team

Yes, team consisted of both permanent and project-financed members No

- 4.34 **Does the WES team deal with other work tasks as well?**

Yes, the team members deal with other tasks Yes, part of the team deal with other tasks

No, the team is fully dedicated to the WES system

# System mapping and baseline information

Objectives of the System Mapping exercise:

EU-WISH is paved around two ambitious aims:

***"Enhancing, extending and consolidating national wastewater surveillance for public health" and "Collaboration and sharing to avoid duplication of efforts and to ensure synergies, based on the development of collaborative structures to enable the development of WES globally"***

However, these two objectives might sound vague. How can we really do that?

To facilitate the progress, evaluation and success of EU-WISH, we have developed nine Key Performance Indicators (KPIs) that will provide us with tangible and clear objectives to achieve by October 2026.

The baseline questions related to these KPIs have been structured in the three following sections of the survey:

**Section 5.1** includes the first two KPIs measuring the effectiveness of EU-WISH consolidating WES strategies at national and international (cross-border) level.

**Section 5.2** focuses on enhancing and extending national capacities for WES. Are WES capacities by the end of 2026 larger than at the beginning of 2024? Are participants followings the recommendations from EU-WISH technical WPs?

**Section 5.3** is focused on the dissemination efforts expected to increase the awareness and engagement of the international community with the project.

The baseline information collected in this system mapping exercise will help us to:

1. **take informed decisions to promote and support the institutionalization of WES in areas and for pathogens, including other public health related targets, of interest.**
2. **to assess and evaluate the progress and success of the EU-WISH Joint Action.**

### WES Strategies

Information KPI 1: *Number of national wastewater and environmental surveillance (WES) strat egies* ***implemented*** *and* ***integrated*** *at national level with other types of surveillance indicating where relevant if these are used to informed decision making.*

With the following questions, we need to identify the number of strategies implemented and integrated at the beginning of EU-WISH in order to monitor and achieve the implementation of at least 10 additional strategies by October 2026.

**"*PAST*"** (previous operative systems) will give us an idea of the trajectory of WES within participating countries. Some public health institutions could be having financial cuts that could negatively influence the operational status of their WES systems in the short term.

**"PRESENT"** will collect the baseline information needed to consolidate and enhance WES capacities and track the progress during the Joint Action. Additional and more granular information will be collected through pathogen- specific sections (6-12).

**"*FUTURE*"** instead, will help us identify participating countries that are very close to implement WES, being potential "low-hanging fruit" targets for our KPI number 1.

*PAST*

- 5.1.1 **Did you have any operative WES system in your country before January 1st, 2024.**

Yes No

- 5.1.2 **Please select your operative WES systems before January 1st, 2024.**

**Emerging pathogens:** Mpox, Dengue, WestNile, Zika, Chikungunya, Viral hemorrhagic fevers, Nipah / Hendra, Candida auris...

SARS-CoV-2

Influenza or other respiratory viruses

Polio and other non-polio enteroviruses (NPEV) Antimicrobial resistance (AMR)

Emerging pathogens Illicit drugs

Chemicals and health-related biomarkers Others

- - 1. **Please specify others WES systems.**
    2. **If one or several of the WES systems are not operative anymore, please provide the reason**

*PRESENT*

- 5.1.5 **Do you have an operative WES system in your country in 2024?**

**Jan-May 2024**

Yes No

- 5.1.6 **Please select the targets of your operative WES system in 2024**

***Emerging pathogens****: Mpox, Dengue, WestNile, Zika, Chikungunya, Viral hemorrhagic fevers, Nipah / Hendra, Candida auris...*

SARS-CoV-2

Influenza or other respiratory viruses

Polio and other non-polio enteroviruses (NPEV) Antimicrobial resistance (AMR)

Emerging pathogens Illicit drugs

Chemicals and health-related biomarkers Others

5.1.7 Please specify other targets in your WES system.

*FUTURE*

- 5.1.8 **Are you planning to implement new or additional operative WES system for any of these targets in late 2024 or early 2025?**

***Emerging pathogens****: Mpox, Dengue, WestNile, Zika, Chikungunya, Viral hemorrhagic fevers, Nipah / Hendra, Candida auris...*

SARS-CoV-2

Influenza or other respiratory viruses

Polio and other non-polio enteroviruses (NPEV)

Antimicrobial resistance (AMR) Emerging pathogens

Illicit drugs

Chemicals and health-related biomarkers I do not know

No

Yes, others

5.1.9 Yes, other pathogens or substances:

*OVERALL CHALLENGES*

- 5.1.10 **Rank the main challenges implementing WES system in your country or the areas where support would be needed to accelerate the implementation and integration of WES**

*Use drag&drop or the up/down buttons to change the order or accept the initial order.*

**Integration**: translation and integration of the final results into clinical surveillance and response systems

**Data warehouse**: no centralized IT system for wastewater surveillance data storage, access or sharing

**Estimates**: difficulties with data normalization, modelling and interpretation

**Analysis**: verification of methods due to lack of laboratory capacities and/or experience. QA/QC, reference materials/inter-labs

**Operationalization**: consistency in testing frequency and analysis between varying sites and laboratories running the samples

**Sampling**: challenges with the logistics to secure the collection of samples and consequent analysis

**Funding**: lack of financial support to ensure operationalization and sustainability

**Coordination**: lack of coordination among Ministries or other leading organizations

Information KPI 2: *Number of wastewater surveillance strategies developed to address* ***cross- border health threats*** *specific to priority pathogens or relevant substances.*

Here, we aim to identify organizations that could implement cross-border strategies before October 2026.

- 5.1.11 **Has your country, or any territorial unit within your country, used WES to detect cross- border public health threats at points of entry or big international events?**

Yes No

- 5.1.12 **If so, are there any measures implemented in your country based on positive WES results at point of entry?**

Yes No

- 5.1.13 **If so (5.1.11), is the surveillance system at points of entry a permanent feature, or is it activated occasionally in response to specific public health threats?**

Permanent

Activated on occasion

5.1.14 Please specify conditions or triggers for activation

- 5.1.15 **If so, which pathogens or substances do you monitor at these points of entry or big events?**

SARS-CoV-2

Influenza or other respiratory viruses

Polio and other non-polio enteroviruses (NPEV) Antimicrobial resistance (AMR)

Emerging pathogens Illicit drugs

Chemicals and health-related biomarkers Others

5.1.16 Please specify other targets monitored at point of entry.

- 5.1.17 **If so, which types of points of entry are included in the surveillance system?**

Airports/Commercial aircrafts Seaports/Maritime vessels Land borders

Tv2

Public transport/Trains

Big events/International festivals Others

5.1.18 Please specify other types of point of entry included in your WES system.

- 5.1.19 **Did you sign up for the EU supersites program?**

**Find information about the program** [**here**](https://ec.europa.eu/eusurvey/runner/EU4S_SuperSites)

Yes No

### Capacities at national level

#### Information KPI 3, 4 and 5 - Increased WES **capacity** at national level.

- 5.2.1 **For those countries with an operative WES system: who is responsible for the sample collection?**

Water utilities or authorities Public health authorities

Other (please specify)

- - 1. **If other, please specify who is responsible.**
    2. **Frequency: how often do you collect samples for WES at WWTP level in 2024.**

**Please use as reference the period Jan-May 2024.**

|  | No collection | Daily | Multiple per week | Weekly | Monthly | Once per quarter | More than once per year | Once per year | It varies from sampling sites or based on the purpose of the WES system |
| --- | --- | --- | --- | --- | --- | --- | --- | --- | --- |
| SARS-CoV-2 |  |  |  |  |  |  |  |  |  |
| Influenza or other respiratory viruses |  |  |  |  |  |  |  |  |  |
| Polio and other non- polio enteroviruses (NPEV) |  |  |  |  |  |  |  |  |  |
| Antimicrobial resistance (AMR) |  |  |  |  |  |  |  |  |  |
| Emerging pathogens |  |  |  |  |  |  |  |  |  |
| Illicit drugs |  |  |  |  |  |  |  |  |  |
| Chemicals and health- related biomarkers |  |  |  |  |  |  |  |  |  |

- - 1. **What is the approximate monthly average number of samples processed for WES in your country in 2024?**

**Please use as reference Jan-May 2024.**

|  | Average number of samples analyzed per month |
| --- | --- |
| SARS-CoV-2 | ww samples |
| Influenza or other respiratory viruses | ww samples |
| Polio and other non-polio enteroviruses (NPEV) | ww samples |
| Antimicrobial resistance (AMR) | ww samples |
| Emerging pathogens | ww samples |
| Illicit drugs | ww samples |
| Chemicals and health-related biomarkers | ww samples |

- - 1. **Do you have an adaptable/flexible WES sampling strategy in case of a positive detection or emergency (an existing adaptative strategy is in place and documented)**

**e.g.: increasing resolution, or frequency?**

*at most 1 answered row(s)*

|  | Yes | No |
| --- | --- | --- |
| SARS-CoV-2 |  |  |
| Influenza or other respiratory viruses |  |  |
| Polio and NPEV |  |  |
| AMR |  |  |
| Emerging pathogens |  |  |
| Illicit drugs |  |  |
| Chemicals and health-related biomarkers |  |  |

#### 23

- - 1. **Which specific pathogens or substances do you monitor in 2024 (Jan-May) under your national WES monitoring program?**

*e.g. specific SARS-CoV-2 variants, specific ARGs, name of the illicit drugs...*

How many laboratories are involved in the surveillance?

Are you participating in either national or international ring trials?

Have you performed in-silico ring trials on sequencing analysis ("dry lab" tests)?

|  | Name of the targets included and analyzed in your methods in  2024 | Number of laboratories | Participation in interlaboratory/ring  trial? | Performed in-silico ring  trials? |
| --- | --- | --- | --- | --- |
| SARS-CoV-2 |  |  | Yes No | Yes No  I do not know |
| Influenza or other RVs |  |  | Yes No | Yes No  I do not know |
| Polio and other enteroviruses |  |  | Yes No | Yes No  I do not know |
| AMR |  |  | Yes No | Yes No  I do not know |
| Emerging pathogens |  |  | Yes No | Yes No  I do not know |

| Illicit drugs |  |  | Yes No | Yes No  I do not know |
| --- | --- | --- | --- | --- |
| Chemicals and health-related biomarkers |  |  | Yes No | Yes No  I do not know |

- 5.2.7 **Fraction of the population covered by WWTPs?** *(in %) Percent of your country’s population connected to sewer systems*

%

- 5.2.8 **What is the total number of WWTPs in your country?**

5.2.9 Number of WWTPs and and estimated number of inhabitants served by the sewerage network within your current WES system?

|  | Number of WWTPs monitoring each target | Approximate population served |
| --- | --- | --- |
| SARS-CoV-2 | WWTPs | inhabitants |
| Influenza or other RVs | WWTPs | inhabitants |
| Polio and NPEV | WWTPs | inhabitants |
| AMR | WWTPs | inhabitants |
| Emerging pathogens | WWTPs | inhabitants |
| Illicit drugs | WWTPs | inhabitants |
| Chemicals and health-related biomarkers | WWTPs | inhabitants |

- 5.2.10 **Please rank the following aspects considered when prioritizing the spatial and temporal resolution of your WES system**

**i.e., selections of sites**

*Use drag&drop or the up/down buttons to change the order or accept the initial order.*

Legal issues

Management issues (coordination, involved authorities)

Added value to inform public health decision making

Resources and Technical Feasibility

Population coverage

- - 1. **Any other types of WES sampling strategy implemented in your country in 2024? Have you used or developed ethical guidelines before utilizing any of these approaches?**

|  | Sampling Strategy | | Ethical guidelines  developed |
| --- | --- | --- | --- |
| SARS-CoV-2 | No Hospitals  Nursing homes  Building level | Manholes Transport hubs Big events  No-sewer (open channels) | Yes No |
| Influenza or other RVs | No Hospitals  Nursing homes  Building level | Manholes Transport hubs Big events  No-sewer (open channels) | Yes No |
| Polio and NPEV | No Hospitals  Nursing homes  Building level | Manholes Transport hubs Big events  No-sewer (open channels) | Yes No |
| AMR | No Hospitals  Nursing homes  Building level | Manholes Transport hubs Big events  No-sewer (open channels) | Yes No |
| Emerging pathogens | No Hospitals  Nursing homes  Building level | Manholes Transport hubs Big events  No-sewer (open channels) | Yes No |
| Illicit drugs | No Hospitals  Nursing homes  Building level | Manholes Transport hubs Big events  No-sewer (open channels) | Yes No |

| Chemicals and health-related biomarkers | No Manholes  Hospitals Transport hubs  Nursing Big events homes  Building level No-sewer (open  channels) | Yes No |
| --- | --- | --- |

- - 1. **Further Details.**

Would you be willing to provide additional information regarding your country's sampling strategy in a subsequent questionnaire?

If so, please provide a contact email below.

### Awareness and engagement

Information KPI 6, 7, 8 and 9: ***Awareness****, stakeholder* ***engagement*** *and activities opened with* ***LMICs.***

- - 1. **Is your WES data used for public awareness?**

**i.e., website, dashboards**

|  | Y/N | | Type of content | Please add the link if possible |
| --- | --- | --- | --- | --- |
| SARS-CoV-2 | Yes | No | Website  Dashboard Report/PDF  Regular media bulletin Other |  |
| Influenza or other RVs | Yes | No | Website  Dashboard Report/PDF  Regular media bulletin Other |  |
| Polio and NPEV | Yes | No | Website  Dashboard Report/PDF  Regular media bulletin Other |  |
| AMR | Yes | No | Website  Dashboard Report/PDF  Regular media bulletin Other |  |
| Illicit drugs | Yes | No | Website  Dashboard Report/PDF  Regular media bulletin Other |  |

| Emerging pathogens | Yes No | Website  Dashboard Report/PDF  Regular media bulletin Other |  |
| --- | --- | --- | --- |
| Chemicals and health-related biomarkers | Yes No | Website  Dashboard Report/PDF  Regular media bulletin Other |  |

- - 1. **Please specify other type of content for the specific pathogen and link if possible.**

#### 31

- - 1. **Who is your reporting intended to?**

|  | Scientific community | Decision- makers | Our own group  /institution | General public | Other authorities |
| --- | --- | --- | --- | --- | --- |
| SARS-CoV-2 |  |  |  |  |  |
| Influenza or other RVs |  |  |  |  |  |
| Polio and NPEV |  |  |  |  |  |
| AMR |  |  |  |  |  |
| Emerging pathogens |  |  |  |  |  |
| Illicit drugs |  |  |  |  |  |
| Chemicals and health- related biomarkers |  |  |  |  |  |

#### 32

- - 1. **Are there dedicated channels to communicate sensitive data?**

**e.g. illegal substances to the public safety authorities?**

|  | Yes | No |
| --- | --- | --- |
| SARS-CoV-2 |  |  |
| Influenza or other RVs |  |  |
| Polio and NPEV |  |  |
| AMR |  |  |
| Emerging pathogens |  |  |
| Illicit drugs |  |  |
| Chemicals and health-related biomarkers |  |  |

- - 1. **If so, what kind of channels?**
- 5.3.6 **Are you planning to organize any learning or communication activity (seminar, workshop, media interview, conference) to increase the awareness of WES in 2024?**

Yes (*please reach out WP2 and WP4 before that happens!*) No

- 5.3.7 **Are you collaborating with organizations at Low- and Middle-income Countries (LMICs) for WES?**

**e.g. through research projects/collaborations,** [**see example**](https://cordis.europa.eu/project/id/101103253)

Yes, and I can share the details of the organizations and projects below No, I don't have any current collaboration

Not in this context, but I can share the details of other organizations from LMICs that we are collaborating or are part of our network

5.3.8 Could you name those organizations?

- 5.3.9 **Are you currently engaging with international stakeholders within the framework of your national WES system 2024?**

***i.e., The World Bank***

Yes No

5.3.10 Could you name those stakeholders?

- 5.3.11 **Do you have WES training material that could be shared with the rest of the consortium?**

**click yes to see more options**

Yes No

- 5.3.12 **Types of training material available**

*(pdf, Youtube, other platforms e.g. ECDC Virtual Academy - EVA)*

National report: experience establishing pilot project Sampling protocols

Analytical procedures for detection and quantification of different targets Data platform/visualization

Statistical methods

Engagement with policymakers

Integration into Public Health systems/decisionmakers

# SARS-CoV-2

Definition of "**operative WES system**":

*Wastewater and environmental surveillance program implemented 1) either at research level or national public health level, 2) with either limited duration or institutionalized, 3) where data is either in exploratory phase or reported to key decision-makers.*

- 6.1 **Do you have an operative WES system for SARS-CoV-2 in your country in 2024?**

Yes No

- 6.2 **What is the main governmental SARS-CoV-2 WES authority in your country?**

**Ministry of Health, Ministry of Interior, National Health Institute**

- 6.3 **Who is the main contact point for the governmental SARS-CoV-2 WES authority in your country?**

**Name of contact point**

- 6.4 **Name of the organizations and main contact points coordinating and involved in the SARS-CoV- 2 WES system?**

**Name of organization, name of the main contact point**

- 6.5 **Since when have you applied WES for SARS-CoV-2?**

2020

2021

2022

2023

2024

- 6.6 **How is the SARS-CoV-2 WES currently implemented in your country?**

**Jan-May 2024**

As a research project with limited duration, *e.g. exploring possibilities*

As a public health project with limited duration, *e.g. piloting routine surveillance*

Already institutionalized, *e.g. routine surveillance with unlimited runtime and stable funding*

Other

6.7 Other implementation

- 6.8 **End of the financial support for the WES system?**

**Please write the date in such case.**

- 6.9 **What are the main objectives of the SARS-CoV-2 WES system in your country?**

Early detection Trends

Variants

- 6.10 **Is the SARS-CoV-2 WES system integrated with other types of SARS-CoV-2 surveillance indicators in your country?**

Yes, fully integrated with multiple other indicators Yes, fully integrated with one indicator

Yes, partially integrated using several indicators No

6.11 If so, which indicators and what is the main objective?

**i.e., laboratory confirmed COVID-19 cases, variants detected from the clinical samples, hospitalizations, other (free text).**

- 6.12 **Is SARS-CoV-2 WES used for public health decision making in your country?**

Yes

I don't know No

- 6.13 **If so, since when?**

2020

2021

2022

2023

2024

- 6.14 **If so, how?**

**How are results aggregated, presented, how often reported and how are interpreted; epidemiologically or statistically?**

- 6.15 **Is there a long-term strategy for SARS-CoV-2 WES in your country?**

Yes No

- 6.16 **Is there a data ownership policy for the SARS-CoV-2 WES system in your country?**

Yes No

- 6.17 **Who owns the raw and processed data?**

Water utilities own the data

Public health institutions own the data

Laboratory/Service providers own the data Open data

Municipalities own the data University or research center I don't know

Other

- 1. **Other or additional relevant information about data ownership.**
  2. **Rate the main challenges for the SARS-CoV-2 WES system in your country or the areas where support would be most needed.**
  - **Not challenging**

****Slightly Challenging**

*****Moderately Challenging**

******Very Challenging**

*******Extremely Challenging**

| **Coordination**: lack of coordination among Ministries or other leading organizations |  |
| --- | --- |
| **Funding**: lack of financial support to ensure operationalization and sustainability |  |
| **Sampling**: challenges with the logistics to secure the collection of samples and consequent analysis |  |
| **Operationalization**: consistency in testing frequency and analysis between varying sites and laboratories running the samples |  |
| **Analysis**: verification of methods due to lack of laboratory capacities and/or experience. QA  /QC, reference materials/inter-labs |  |
| **Estimates**: difficulties with data normalization, modelling and interpretation |  |
| **Data warehouse**: no centralized IT system for wastewater surveillance data storage, access or sharing |  |
| **Integration**: translation and integration of the final results into clinical surveillance and response systems |  |

# Influenza and other respiratory viruses (RVs)

Definition of "**operative WES system**":

*Wastewater and environmental surveillance program implemented 1) either at research level or national public health level, 2) with either limited duration or institutionalized, 3) where data is either in exploratory phase or reported to key decision-makers.*

- 7.1 **Do you have an operative WES system for influenza and/or RVs in your country in 2024?**

Yes No

- 7.2 **What is the main governmental Influenza and/or RVs WES authority in your country?**

**Name of authority**

- 7.3 **Who is the main contact point for the governmental Influenza and/or RVs WES authority in your country?**

**Name of contact point**

- 7.4 **Name of the organizations and main contact points coordinating and involved in the Influenza and/or RVs WES system?**

**Name of organization, name of the main contact point**

- 7.5 **Since when have you applied WES for Influenza?**

| 2010 or before | 2015 | 2020 |
| --- | --- | --- |
| 2011 | 2016 | 2021 |
| 2012 | 2017 | 2022 |
| 2013 | 2018 | 2023 |
| 2014 | 2019 | 2024 |

- 7.6 **How is the Influenza WES currently implemented in your country?**

**Jan-May 2024**

As a research project with limited duration, *e.g. exploring possibilities*

As a public health project with limited duration, *e.g. piloting routine surveillance*

Already institutionalized, *e.g. routine surveillance with unlimited runtime and stable funding*

Other

7.7 Other implementation

- 7.8 **End of the financial support for the WES system?**

**Please write the date.**

- 7.9 **What are the main objectives of the Influenza and/or RVs WES system in your country?**

Early detection Trends

Variants

- 7.10 **Is the Influenza WES system integrated with other types of surveillance indicators in your country?**

Yes, multiple other indicators Yes, one other indicator

No

7.11 If so, which indicators

- 7.12 **Is Influenza WES used for public health decision making in your country?**

Yes No

- 7.13 **If so, since when?**

| 2010 | 2015 | 2020 |
| --- | --- | --- |
| 2011 | 2016 | 2021 |
| 2012 | 2017 | 2022 |
| 2013 | 2018 | 2023 |
| 2014 | 2019 | 2024 |

- 7.14 **If so, how?**

How are results aggregated, presented, how often reported and how are interpreted; epidemiologically or statistically

- 7.15 **Is there a long-term strategy for Influenza WES in your country?**

Yes No

7.16 Is there any other respiratory virus (RV) analyzed in your WES system?

**Please write the pathogen, how the WES system is implemented, name of the organizations and if the results are combined with other indicators and used by decision makers.**

- 7.17 **Is there a data ownership policy?**

Yes No

- 7.18 **Who owns the raw and processed data?**

Water utilities own the data

Public health institutions own the data

Laboratory/Service providers own the data Open data

Municipalities own the data University or research center I don't know

Other

- 1. **Other or additional relevant information about data ownership.**
  2. **Rate the main challenges for the Influenza and/or RVs WES system in your country or the areas where support would be most needed.**
  - **Not challenging**

****Slightly Challenging**

*****Moderately Challenging**

******Very Challenging**

*******Extremely Challenging**

| **Coordination**: lack of coordination among Ministries or other leading organizations |  |
| --- | --- |
| **Funding**: lack of financial support to ensure operationalization and sustainability |  |
| **Sampling**: challenges with the logistics to secure the collection of samples and consequent analysis |  |
| **Operationalization**: consistency in testing frequency and analysis between varying sites and laboratories running the samples |  |

| **Analysis**: verification of methods due to lack of laboratory capacities and/or experience. QA  /QC, reference materials/inter-labs |  |
| --- | --- |
| **Estimates**: difficulties with data normalization, modelling and interpretation |  |
| **Data warehouse**: no centralized IT system for wastewater surveillance data storage, access or sharing |  |
| **Integration**: translation and integration of the final results into clinical surveillance and response systems |  |

# Poliovirus (PV) and non-polio enteroviruses (NPEVs)

Definition of "**operative WES system**":

*Wastewater and environmental surveillance program implemented 1) either at research level or national public health level, 2) with either limited duration or institutionalized, 3) where data is either in exploratory phase or reported to key decision-makers.*

- 8.1 **Do you have an operative WES system for PV and/or NPEVs in your country in 2024?**

Yes No

- 8.2 **What is the main governmental PV and NPEVs WES authority in your country?**

**Name of authority**

- 8.3 **Who is the main contact point for the governmental PV and NPEVs WES authority in your country?**

**Name of contact point**

- 8.4 **Name of the organizations and main contact points coordinating and involved in the PV and/or NPEVs WES system?**

**Name of organization, name of the main contact point**

- 8.5 **Since when have you applied WES for Polio?**

| 2010 or before | 2015 | 2020 |
| --- | --- | --- |
| 2011 | 2016 | 2021 |
| 2012 | 2017 | 2022 |
| 2013 | 2018 | 2023 |
| 2014 | 2019 | 2024 |

- 8.6 **Is you national Polio WES supporting the WHO Global Polio Eradication Initiative (GPEI) program i.e., by providing complementary surveillance for the European Regional Commission for the Certification of Poliomyelitis Eradication (RCC)**

[https://www.who.int/europe/groups/european-regional-commission-for-the-certification-of-poliomyelitis-eradication-rcc](http://www.who.int/europe/groups/european-regional-commission-for-the-certification-of-poliomyelitis-eradication-rcc)

Yes No

- 8.7 **What is the main reason for the existence of the Polio WES system in your country?**

My country is classified with intermediate risk of Poliovirus reintroduction To take part to the GPEI

To simply gain useful and complementary information in regards to PV and NPEV For research purposes

Others: please specify

- 1. **Other reasons**
  2. **What are the reason for being classified with intermediate risk**

Non-sufficient quality surveillance Low vaccination coverage

Presence of high-risk populations/ areas

Presence of Poliovirus-essential facility (PEF) Other: specify

- 1. **Other reason**
- 8.11 **How is the Polio WES currently implemented and funded in your country?**

**Jan-May 2024**

As a research project with limited duration, *e.g. exploring possibilities*

As a public health project with limited duration and funding, *e.g. piloting routine surveillance*  Already institutionalized, *e.g. routine surveillance with unlimited runtime and stable funding*  Other

8.12 Other implementation

- 8.13 **It is funded at national, regional, or local level? Name of the funding agency?**
- 8.14 **End of the financial support for the WES system?**

**Please write the date.**

- 8.15 **Have you developed guidelines or regulatory documents for using WES data for PV in your country?**

See example CDC:

<https://www.cdc.gov/polio/php/laboratories/wastewater-testing-considerations.html>

Yes No

8.16 If so, who is the main actor/institution responsible to decide on the Polio WES actions, in case of a WES positive sample is found?

- 8.17 **Is your main National Public Health Institution, legally responsible for the good management of Polio surveillance, receiving the relevant WES Polio results allowing it to take proper actions?**

Yes No

- 8.18 **Is there a long-term strategy for Polio WES in your country?**

Yes No

- 8.19 **Is the Polio WES system integrated with other types of surveillance indicators in your country?**

Yes, multiple other indicators Yes, one other indicator

No

8.20 If so, which indicators

- 8.21 **Is Polio WES used for public health decision making in your country?**

Yes

No

- 8.22 **If so, since when?**

| 2010 or before | 2015 | 2020 |
| --- | --- | --- |
| 2011 | 2016 | 2021 |
| 2012 | 2017 | 2022 |
| 2013 | 2018 | 2023 |
| 2014 | 2019 | 2024 |

- 8.23 **If so, how?**

How are results aggregated, presented, how often reported and how are interpreted; epidemiologically or statistically?

- 8.24 **On which criteria did you base (or intend to base) the epidemiological strategy of your sampling scheme?**

Areas presenting high-risk populations

Areas where a PEF (Polio Essential Facility) is present Big cities for a good population coverage

Other

8.25 **Other**

- 8.26 **Consideration of global migrations and its integration into WES Polio (asylum shelters): do you have a system in place to take into account global migrations for sampling site selection**

/frequency?

Yes No

- 8.27 **Is there a data ownership policy?**

Yes No

- 8.28 **Who owns the raw and processed data?**

Water utilities own the data

Public health institutions own the data

Laboratory/Service providers own the data Open data

Municipalities own the data University or research center

I don't know Other

- 1. **Other or additional relevant information about data ownership.**
  2. **Rate the main challenges for the Polio WES system in your country or the areas where support would be most needed.**
  - **Not challenging**

****Slightly Challenging**

*****Moderately Challenging**

******Very Challenging**

*******Extremely Challenging**

| **Coordination**: lack of coordination among Ministries or other leading organizations |  |
| --- | --- |
| **Funding**: lack of financial support to ensure operationalization and sustainability |  |
| **Sampling**: challenges with the logistics to secure the collection of samples and consequent analysis |  |
| **Operationalization**: consistency in testing frequency and analysis between varying sites and laboratories running the samples |  |
| **Analysis**: verification of methods due to lack of laboratory capacities and/or experience. QA  /QC, reference materials/inter-labs |  |
| **Estimates**: difficulties with data normalization, modelling and interpretation |  |
| **Data warehouse**: no centralized IT system for wastewater surveillance data storage, access or sharing |  |
| **Integration**: translation and integration of the final results into clinical surveillance and response systems |  |

Non-polio enteroviruses

- 8.31 **Do you have a national public health surveillance system for NPEV?**

Yes No

- 8.32 **Is there any NPEVs analyzed in your WES system?**

Yes No

- 8.33 **If you are performing NPEV WES, do you have a system in place to take into account global migrations for selecting specific NPEV?**

Yes No

- 8.34 **Do you screen for Polio and/or NPEV at point of entry level (i.e., airport)?**

Yes No

8.35 Where are the major needs in your country in terms of gaps to be filled in, or knowledge

/experience to acquire:

*Use drag&drop or the up/down buttons to change the order or accept the initial order.*

Having clear guidelines

Having a strategy in tackling (or not) PEF (Polio Essential Facilities), such as vaccine producers, within the surveillance

Being able to react properly if a positive sample is found in WES (outbreak response plan)

Convincing authorities of the interest of financing a Polio and NPEV WES

(Beyond using NPEV as a quality control,) having a list of NPEV targets to specifically screen

#### AMR

Definition of "**operative WES system**":

*Wastewater and environmental surveillance program implemented 1) either at research level or national public health level, 2) with either limited duration or institutionalized, 3) where data is either in exploratory phase or reported to key decision-makers.*

- 9.1 **Do you have an operative WES system for AMR in your country in 2024?**

Yes No

- 9.2 **What is the main governmental AMR WES authority in your country?**

**Name of authority**

- 9.3 **Who is the main contact point for the governmental AMR WES authority in your country?**

**Name of contact point**

- 9.4 **Name of the organizations and main contact points coordinating and involved in the AMR WES system?**

**Name of authority, name of the main contact point**

- 9.5 **Since when have you applied WES for AMR?**

| 2010 or before | 2015 | 2020 |
| --- | --- | --- |
| 2011 | 2016 | 2021 |
| 2012 | 2017 | 2022 |
| 2013 | 2018 | 2023 |
| 2014 | 2019 | 2024 |

- 9.6 **How is the AMR WES currently implemented in your country?**

**Jan-May 2024**

As a research project with limited duration, *e.g. exploring possibilities*

As a public health project with limited duration, *e.g. piloting routine surveillance*

Already institutionalized, *e.g. routine surveillance with unlimited runtime and stable funding*

Other

9.7 Other implementation

- 9.8 **End of the financial support for the WES system?**

**Please write the date.**

- 9.9 **What are the main objectives of the AMR WES system in your country?**

Early detection Trends

Variants Other

- 1. **Other implementation**
  2. **Do you currently perform or did you perform in previous years WES for AMR?**

|  | Yes/No | Start year | End year or currently (select 2024) | Frequency | Detection type | Detection technics? |
| --- | --- | --- | --- | --- | --- | --- |
| ESBL  (beta-lactamase E. coli) | Yes Yes No No |  |  | Daily  Multiple per week Weekly  Monthly  Once per quarter  More than once per year Once per year  Variable | Bacteria ARGs | Culture of resistant bacteria WGS of culture  qPCR for resistance genes Metagenome  Other |
| CPE  (carbapenase enterobacter) | Yes Yes No No |  |  | Daily  Multiple per week Weekly  Monthly  Once per quarter  More than once per year Once per year  Variable | Bacteria ARGs | Culture of resistant bacteria WGS of culture  qPCR for resistance genes Metagenome  Other |
| VRE  (vancomycin resistant Enterococcus) | Yes Yes No No |  |  | Daily  Multiple per week Weekly  Monthly  Once per quarter  More than once per year Once per year  Variable | Bacteria ARGs | Culture of resistant bacteria WGS of culture  qPCR for resistance genes Metagenome  Other |

| Plasmids or mobile genetic elements | Yes Yes No No |  |  | Daily  Multiple per week Weekly  Monthly  Once per quarter  More than once per year Once per year  Variable | Bacteria ARGs | Culture of resistant bacteria WGS of culture  qPCR for resistance genes Metagenome  Other |
| --- | --- | --- | --- | --- | --- | --- |
| Panel of Resistance genes | Yes Yes No No |  |  | Daily  Multiple per week Weekly  Monthly  Once per quarter  More than once per year Once per year  Variable | Bacteria ARGs | Culture of resistant bacteria WGS of culture  qPCR for resistance genes Metagenome  Other |
| tetracycline resistant gens | Yes Yes No No |  |  | Daily  Multiple per week Weekly  Monthly  Once per quarter  More than once per year Once per year  Variable | Bacteria ARGs | Culture of resistant bacteria WGS of culture  qPCR for resistance genes Metagenome  Other |
| Suplphonamide resistant gens | Yes Yes No No |  |  | Daily  Multiple per week Weekly  Monthly  Once per quarter  More than once per year Once per year  Variable | Bacteria ARGs | Culture of resistant bacteria WGS of culture  qPCR for resistance genes Metagenome  Other |

| 3rd-generation cephalosporin resistant gens | Yes Yes No No |  |  | Daily  Multiple per week Weekly  Monthly  Once per quarter  More than once per year Once per year  Variable | Bacteria ARGs | Culture of resistant bacteria WGS of culture  qPCR for resistance genes Metagenome  Other |
| --- | --- | --- | --- | --- | --- | --- |
| Beta-lactam resistance genes | Yes Yes No No |  |  | Daily  Multiple per week Weekly  Monthly  Once per quarter  More than once per year Once per year  Variable | Bacteria ARGs | Culture of resistant bacteria WGS of culture  qPCR for resistance genes Metagenome  Other |
| Quinolone resistance genes | Yes Yes No No |  |  | Daily  Multiple per week Weekly  Monthly  Once per quarter  More than once per year Once per year  Variable | Bacteria ARGs | Culture of resistant bacteria WGS of culture  qPCR for resistance genes Metagenome  Other |
| Macrolide resistance genes | Yes Yes No No |  |  | Daily  Multiple per week Weekly  Monthly  Once per quarter  More than once per year Once per year  Variable | Bacteria ARGs | Culture of resistant bacteria WGS of culture  qPCR for resistance genes Metagenome  Other |

| Aminoglycoside resistance genes | Yes Yes No No |  |  | Daily  Multiple per week Weekly  Monthly  Once per quarter  More than once per year Once per year  Variable | Bacteria ARGs | Culture of resistant bacteria WGS of culture  qPCR for resistance genes Metagenome  Other |
| --- | --- | --- | --- | --- | --- | --- |

- 1. **Information about type of program and funding**

|  | Type program  **Jan-May 2024** | Funding | Aim of the program | Main contact email |
| --- | --- | --- | --- | --- |
| ESBL | As a research project with limited duration, e.g. exploring possibilities As a public health project with limited duration and funding, e.g. piloting  routine surveillance  Already institutionalized, e.g. routine surveillance with unlimited runtime and stable funding  Other | Self-funded  National or international research funding  Ministry funding Other |  |  |
| CPE | As a research project with limited duration, e.g. exploring possibilities As a public health project with limited duration and funding, e.g. piloting  routine surveillance  Already institutionalized, e.g. routine surveillance with unlimited runtime and stable funding  Other | Self-funded  National or international research funding  Ministry funding Other |  |  |
| VRE | As a research project with limited duration, e.g. exploring possibilities As a public health project with limited duration and funding, e.g. piloting  routine surveillance  Already institutionalized, e.g. routine surveillance with unlimited runtime and stable funding  Other | Self-funded  National or international research funding  Ministry funding Other |  |  |
| Plasmids or mobile genetic elements | As a research project with limited duration, e.g. exploring possibilities As a public health project with limited duration and funding, e.g. piloting  routine surveillance  Already institutionalized, e.g. routine surveillance with unlimited runtime and stable funding  Other | Self-funded  National or international research funding  Ministry funding Other |  |  |

| Panel of Resistance genes | As a research project with limited duration, e.g. exploring possibilities As a public health project with limited duration and funding, e.g. piloting  routine surveillance  Already institutionalized, e.g. routine surveillance with unlimited runtime and stable funding  Other | Self-funded  National or international research funding  Ministry funding Other |  |  |
| --- | --- | --- | --- | --- |
| Tetracycline resistant gens | As a research project with limited duration, e.g. exploring possibilities As a public health project with limited duration and funding, e.g. piloting  routine surveillance  Already institutionalized, e.g. routine surveillance with unlimited runtime and stable funding  Other | Self-funded  National or international research funding  Ministry funding Other |  |  |
| Suplphonamide resistant gens | As a research project with limited duration, e.g. exploring possibilities As a public health project with limited duration and funding, e.g. piloting  routine surveillance  Already institutionalized, e.g. routine surveillance with unlimited runtime and stable funding  Other | Self-funded  National or international research funding  Ministry funding Other |  |  |
| 3rd-generation cephalosporin resistant gens | As a research project with limited duration, e.g. exploring possibilities As a public health project with limited duration and funding, e.g. piloting  routine surveillance  Already institutionalized, e.g. routine surveillance with unlimited runtime and stable funding  Other | Self-funded  National or international research funding  Ministry funding Other |  |  |
| Beta-lactam resistance genes | As a research project with limited duration, e.g. exploring possibilities As a public health project with limited duration and funding, e.g. piloting  routine surveillance  Already institutionalized, e.g. routine surveillance with unlimited runtime and stable funding  Other | Self-funded  National or international research funding  Ministry funding Other |  |  |

| Quinolone resistance genes | As a research project with limited duration, e.g. exploring possibilities As a public health project with limited duration and funding, e.g. piloting  routine surveillance  Already institutionalized, e.g. routine surveillance with unlimited runtime and stable funding  Other | Self-funded  National or international research funding  Ministry funding Other |  |  |
| --- | --- | --- | --- | --- |
| Macrolide resistance genes | As a research project with limited duration, e.g. exploring possibilities As a public health project with limited duration and funding, e.g. piloting  routine surveillance  Already institutionalized, e.g. routine surveillance with unlimited runtime and stable funding  Other | Self-funded  National or international research funding  Ministry funding Other |  |  |
| Aminoglycoside resistance genes | As a research project with limited duration, e.g. exploring possibilities As a public health project with limited duration and funding, e.g. piloting  routine surveillance  Already institutionalized, e.g. routine surveillance with unlimited runtime and stable funding  Other | Self-funded  National or international research funding  Ministry funding Other |  |  |

- 1. **If you have other AMR target, please describe**
- 9.14 **Are the results being related as complementary to epidemiological data from other AMR surveillance systems?**

Yes, interpretation for improvement of treatment guidelines for clinical patients Yes, related with other indicators

No

9.15 If so, which indicators

- 9.16 **Is AMR WES used for public health decision making in your country?**

Yes No

- 9.17 **If so, since when?**

| 2010 or before | 2015 | 2020 |
| --- | --- | --- |
| 2011 | 2016 | 2021 |
| 2012 | 2017 | 2022 |
| 2013 | 2018 | 2023 |
| 2014 | 2019 | 2024 |

- 9.18 **If so, how?**

How are results aggregated, presented, how often reported and how are interpreted; epidemiologically or statistically?

- 9.19 **Is there a long-term strategy for AMR WES in your country?**

Yes No

- 9.20 **Is there a data ownership policy?**

Yes No

- 9.21 **Who owns the raw and processed data?**

Water utilities own the data

Public health institutions own the data

Laboratory/Service providers own the data Open data

Municipalities own the data University or research center I don't know

Other

- 1. **Other or additional relevant information about data ownership.**
  2. **Rate the main challenges for the AMR WES system in your country or the areas where support would be most needed.**
  - **Not challenging**

****Slightly Challenging**

*****Moderately Challenging**

******Very Challenging**

*******Extremely Challenging**

| **Coordination**: lack of coordination among Ministries or other leading organizations |  |
| --- | --- |
| **Funding**: lack of financial support to ensure operationalization and sustainability |  |
| **Sampling**: challenges with the logistics to secure the collection of samples and consequent analysis |  |
| **Operationalization**: consistency in testing frequency and analysis between varying sites and laboratories running the samples |  |
| **Analysis**: verification of methods due to lack of laboratory capacities and/or experience. QA  /QC, reference materials/inter-labs |  |
| **Estimates**: difficulties with data normalization, modelling and interpretation |  |
| **Data warehouse**: no centralized IT system for wastewater surveillance data storage, access or sharing |  |
| **Integration**: translation and integration of the final results into clinical surveillance and response systems |  |

# Emerging pathogens

## Emerging pathogens

An emerging (or re-emerging) pathogen of human health concern in wastewater surveillance can be defined as a pathogen that, at the moment of assessment, displays at least one of the following characteristics:

1. Newly appeared in a population, including pathogens of zoonotic origin.
2. Known to occur in a population but rapidly increasing in circulation (e.g. measured as increased incidence or shedding in wastewater).
3. Known to occur in an area but expanding in geographic range.
4. Known to have occurred in a population and area in the past, declined or eradicated, but now reappearing or increasing in circulation or geographic range.
5. Having undergone genetic mutations leading to a possible increase in virulence, transmissibility, or decreased efficacy of available public health control measures. 6. Declared, or under consideration for declaration, as a Public Health Emergency by national or international health organizations.

Furthermore, to be considered as an emerging pathogen of concern for environmental surveillance under Art. 17 of the UWWD [reference to OJEU to be included], a pathogen should preferably be known or reasonably expected to be shed into wastewater through various body fluids. In addition, an emerging pathogen of concern for environmental surveillance in the context of Art. 17 of the UWWD should display features that allow environmental surveillance to provide additional, complementary or supporting information to clinical surveillance, e.g. detection of asymptomatic or subclinical infections, detection at pre- symptomatic stages, etc.

In the context of the EU-WISH project, emerging pathogens falling within the definition and already covered by tasks/sub-tasks other than 5.6 and 7.2.4 of the project (e.g. SARS-CoV-2 or multidrug resistant pathogenic bacteria) are excluded from the activities.

## Operative WES system

Wastewater and environmental surveillance program implemented 1) either at research level or national public health level, 2) with either limited duration or institutionalized, 3) where data is either in exploratory phase or reported to key decision-makers.

- 10.1 **Do you have an operative WES system for Emerging Pathogens in your country in 2024?**

Yes No

- 10.2 **What is the main governmental Emerging Pathogens WES authority in your country?**

**Name of authority**

- 10.3 **Who is the main contact point for the governmental Emerging Pathogen WES authority in your country?**

**Name of contact point**

- 1. **Name of the organizations and main contact points coordinating and involved in the WES system for Emerging Pathogens?**

**Name of organization, name of the main contact point**

|  | Name of the organizations | Name main contact points coordinating  and involved in the WES of Emerging Pathogens |
| --- | --- | --- |
| Mpox |  |  |
| Dengue |  |  |
| West Nile |  |  |
| Chikungunya |  |  |
| Viral hemorrhagic fevers (Ebola, Marburg, Hantaviruses,  RVF, CCHF, Lassa…) |  |  |
| Nipah / Hendra |  |  |
| Candida auris |  |  |

- 1. **Additional coordination information for other emerging pathogens.**

**Please write the name of the pathogen and the organization and contact point coordinating the WES system**

- 1. **Do you currently perform or did you perform in previous years surveillance for an emerging pathogen?**

|  | Yes/No | Start year | End year or currently (select 2024) |
| --- | --- | --- | --- |
| Mpox | Yes No | *Only values of at least 2010 are allowed* | *Only values of at most 2024 are allowed* |
| Dengue | Yes No | *Only values of at least 2010 are allowed* | *Only values of at most 2024 are allowed* |
| West Nile | Yes No | *Only values of at least 2010 are allowed* | *Only values of at most 2024 are allowed* |
| Chikungunya | Yes No | *Only values of at least 2010 are allowed* | *Only values of at most 2024 are allowed* |
| Viral hemorrhagic fevers (Ebola, Marburg, Hantaviruses, RVF, CCHF, Lassa…) | Yes No | *Only values of at least 2010 are allowed* | *Only values of at most 2024 are allowed* |
| Nipah / Hendra | Yes No | *Only values of at least 2010 are allowed* | *Only values of at most 2024 are allowed* |
| Candida auris | Yes No | *Only values of at least 2010 are allowed* | *Only values of at most 2024 are allowed* |

#### 59

- 1. **Other programs for emerging pathogens.**

**Please write name, start year and end year**

- 1. **How is WES of emerging pathogens currently implemented in your country?**

**Jan-May 2024**

|  | As a research project with limited duration,  e.g. exploring possibilities | As a public health project with limited duration,  e.g. piloting routine surveillance | Already institutionalized,  e.g. routine surveillance with unlimited  runtime  and stable funding |
| --- | --- | --- | --- |
| Mpox |  |  |  |
| Dengue |  |  |  |
| West Nile |  |  |  |
| Chikungunya |  |  |  |
| Viral hemorrhagic fevers (Ebola, Marburg, Hantaviruses,  RVF, CCHF, Lassa…) |  |  |  |
| Nipah / Hendra |  |  |  |
| Candida auris |  |  |  |

- 1. **Other implemented emerging pathogens.**

**Please write name of pathogen and type of implementation E.g. campylobacter, norovirus, cryptosporidium etc.**

- 10.10 **End of the financial support for the WES systems?**

**Please write the date.**

- 10.11 **Is the Emerging Pathogens WES system integrated with other types of surveillance indicators in your country?**

Yes, multiple other indicators Yes, one other indicator

No

I don't know

- 1. **If so, which indicators?**
  2. **Considering all the WES systems for emerging pathogens in place in your country in 2024, are WES findings correlated with data from other surveillance systems?**

**(e.g. epidemiological data from clinical surveillance for infectious diseases or drug prescriptions for pharmaceuticals, etc.)?**

|  | Yes/No | If yes, what kind of correlations were detected? (e.g. detection  before/in the absence clinical cases, anticipation of trends, etc.) |
| --- | --- | --- |
| Mpox | Yes No |  |
| Dengue | Yes No |  |
| West Nile | Yes No |  |
| Chikungunya | Yes No |  |
| Viral hemorrhagic fevers  (Ebola, Marburg, Hantaviruses, RVF, CCHF,  Lassa…) | Yes No |  |
| Nipah / Hendra | Yes No |  |
| Candida auris | Yes No |  |

- 1. **Is WES of Emerging Pathogens used for public health decision making in your country? Is it included into national (integrated) surveillance plans implemented by a government or relevant authorities?**

**(e.g. national surveillance plan for vector-borne diseases, national control plan for infectious diseases, etc.)**

|  | Used for decision making | Included in national surveillance plans |
| --- | --- | --- |
| Mpox | Yes No | Yes No |
| Dengue | Yes No | Yes No |
|  |  |  |

| West Nile | Yes No | Yes No |
| --- | --- | --- |
| Chikungunya | Yes No | Yes No |
| Viral hemorrhagic fevers (Ebola, Marburg, Hantaviruses,  RVF, CCHF, Lassa…) | Yes No | Yes No |
| Nipah / Hendra | Yes No | Yes No |
| Candida auris | Yes No | Yes No |

- 10.15 **If so, how? What kind of decisions/actions?**

**Please summarize how results are aggregated, presented, reported and interpreted?**

- 10.16 **Is there a data ownership policy?**

Yes No

- 10.17 **Who owns the raw and processed data?**

Water utilities own the data

Public health institutions own the data

Laboratory/Service providers own the data Open data

Municipalities own the data University or research center I don't know

Other

- 1. **Other or additional relevant information about data ownership.**
  2. **Rate the main challenges for the Emerging pathognes WES system in your country or the areas where support would be most needed.**
  - **Not challenging**

****Slightly Challenging**

*****Moderately Challenging**

******Very Challenging**

*******Extremely Challenging**

| **Coordination**: lack of coordination among Ministries or other leading organizations |  |
| --- | --- |
| **Funding**: lack of financial support to ensure operationalization and sustainability |  |
| **Sampling**: challenges with the logistics to secure the collection of samples and consequent analysis |  |
| **Operationalization**: consistency in testing frequency and analysis between varying sites and laboratories running the samples |  |
| **Analysis**: verification of methods due to lack of laboratory capacities and/or experience. QA  /QC, reference materials/inter-labs |  |
| **Estimates**: difficulties with data normalization, modelling and interpretation |  |
| **Data warehouse**: no centralized IT system for wastewater surveillance data storage, access or sharing |  |
| **Integration**: translation and integration of the final results into clinical surveillance and response systems |  |

# Illicit drugs

Definition of "**operative WES system**":

*Wastewater and environmental surveillance program implemented 1) either at research level or national public health level, 2) with either limited duration or institutionalized, 3) where data is either in exploratory phase or reported to key decision-makers.*

- 11.1 **Do you have an operative WES system for Illicit drugs in your country in 2024?**

Yes No

- 11.2 **What is the main governmental Illicit drugs WES authority in your country?**

**Name of authority (e.g.** [**Reitox**](https://www.emcdda.europa.eu/about/partners/reitox_en) **national focal point)**

- 11.3 **Who is the main contact point for the governmental Illicit drugs WES authority in your country?**

**Name of contact point**

- 11.4 **Name of the organizations and main contact points coordinating and involved in the Illicit drugs WES system?**

**Name of organization, name of the main contact point**

- 11.5 **Since when have you applied WES for Illicit drugs?**

| 2010 or before | 2015 | 2020 |
| --- | --- | --- |
| 2011 | 2016 | 2021 |
| 2012 | 2017 | 2022 |
| 2013 | 2018 | 2023 |
| 2014 | 2019 | 2024 |

- 11.6 **Is you country participating in the 2024 annual monitoring program organized by the SCORE Network and the EU Drug Agency?**

Please also click in the years your country has participated in the past.

[**Link SCORE/EUDA Dashboard**](https://www.emcdda.europa.eu/publications/html/pods/waste-water-analysis_en)

| 2011 | 2015 | 2019 | 2023 |
| --- | --- | --- | --- |
| 2012 | 2016 | 2020 | 2024 |
| 2013 | 2017 | 2021 |  |
| 2014 | 2018 | 2022 |  |

- 11.7 **How is the Illicit drugs WES currently implemented in your country?**

**Jan-May 2024**

As a research project with limited duration, *e.g. exploring possibilities*

As a public health project with limited duration, *e.g. piloting routine surveillance*

Already institutionalized, *e.g. routine surveillance with unlimited runtime and stable funding*

Other

- 1. **Other implementation**
  2. **Please download this spreadsheet to fill in the list of targets used for a) your national monitoring program and b) for research purposes.**
  3. **Save and upload the list adding your two-letter country codes (ISO 3166-1) at the end of the file name**

**Example: EU-WISH Survey List Illict Drugs_NO**

- 11.11 **End of the financial support for the Illicit drugs WES system?**

**Please write the date.**

- 11.12 **Is the Illicit drugs WES system integrated with other types of surveillance indicators in your country?**

Yes, multiple other indicators Yes, one other indicator

No

11.13 **If so, which indicators**

- 11.14 **Is Illicit drugs WES used for public health decision making in your country?**

Yes No

- 11.15 **If so, since when?**

| 2010 or before | 2015 | 2020 |
| --- | --- | --- |
| 2011 | 2016 | 2021 |
| 2012 | 2017 | 2022 |
| 2013 | 2018 | 2023 |
| 2014 | 2019 | 2024 |

- 11.16 **If so, how?**

How are results aggregated, presented, how often reported and how are interpreted; epidemiologically or statistically?

- 11.17 **Is there a long-term strategy for Illicit drugs WES in your country?**

Yes No

- 11.18 **Is there a data ownership policy?**

Yes No

- 11.19 **Who owns the raw and processed data?**

Water utilities own the data

Public health institutions own the data

Laboratory/Service providers own the data Open data

Municipalities own the data University or research center I don't know

Other

- 1. **Other or additional relevant information about data ownership.**
  2. **Rate the main challenges for the Illicit drugs WES system in your country or the areas where support would be most needed.**
  - **Not challenging**

****Slightly Challenging**

*****Moderately Challenging**

******Very Challenging**

*******Extremely Challenging**

| **Coordination**: lack of coordination among Ministries or other leading organizations |  |
| --- | --- |
| **Funding**: lack of financial support to ensure operationalization and sustainability |  |
| **Sampling**: challenges with the logistics to secure the collection of samples and consequent analysis |  |
| **Operationalization**: consistency in testing frequency and analysis between varying sites and laboratories running the samples |  |
| **Analysis**: verification of methods due to lack of laboratory capacities and/or experience. QA  /QC, reference materials/inter-labs |  |
| **Estimates**: difficulties with data normalization, modelling and interpretation |  |
| **Data warehouse**: no centralized IT system for wastewater surveillance data storage, access or sharing |  |
| **Integration**: translation and integration of the final results into clinical surveillance and response systems |  |

# Chemicals and health-related biomarkers

Definition of "**operative WES system**":

*Wastewater and environmental surveillance program implemented 1) either at research level or national public health level, 2) with either limited duration or institutionalized, 3) where data is either in exploratory phase or reported to key decision-makers.*

- 12.1 **Do you have an operative WES system for Chemicals and health-related biomarkers in your country in 2024?**

Yes No

- 12.2 **What is the main governmental Chemicals and health-related biomarkers WES authority in your country?**

**Name of authority (e.g.** [**Reitox**](https://www.emcdda.europa.eu/about/partners/reitox_en) **national focal point)**

- 12.3 **Who is the main contact point for the governmental Chemicals and health-related biomarkers WES authority in your country?**

**Name of contact point**

- 12.4 **Name of the organizations and main contact points coordinating and involved in the Chemicals and health-related biomarkers WES system?**

**Name of organization, name of the main contact point**

- 12.5 **Since when have you applied WES for Chemicals and health-related biomarkers?**

| 2010 or before | 2015 | 2020 |
| --- | --- | --- |
| 2011 | 2016 | 2021 |
| 2012 | 2017 | 2022 |
| 2013 | 2018 | 2023 |
| 2014 | 2019 | 2024 |

- 12.6 **How is the Chemicals and health-related biomarkers WES currently implemented in your country?**

**Jan-May 2024**

As a research project with limited duration, *e.g. exploring possibilities*

As a public health project with limited duration, *e.g. piloting routine surveillance*

Already institutionalized, *e.g. routine surveillance with unlimited runtime and stable funding*

Other

- 1. **Other implementation**
  2. **Please download this spreadsheet to fill in the list of targets used for a) your national monitoring program and b) for research purposes.**

[EU-WISH_Survey._List_Chemicals_and_HR_biomarkers.xlsx](https://ec.europa.eu/eusurvey/files/78cf212e-7aea-4802-888b-114529e1006c/50a49916-6a03-40ce-8a6c-bdcefadceb00)

- 1. **Save and upload the list adding your two-letter country codes (ISO 3166-1) at the end of the file name**

**Example: EU-WISH Survey List Chemicals nad HR biomarkers_NO**

- 12.10 **End of the financial support for the Chemicals and health-related biomarkers WES system?**

**Please write the date.**

- 12.11 **Is the Chemicals and health-related biomarkers WES system integrated with other types of surveillance indicators in your country?**

Yes, multiple other indicators Yes, one other indicator

No

12.12 **If so, which indicators**

- 12.13 **Is Chemicals and health-related biomarkers WES used for public health decision making in your country?**

Yes No

- 12.14 **If so, since when?**

| 2010 or before | 2015 | 2020 |
| --- | --- | --- |
| 2011 | 2016 | 2021 |
| 2012 | 2017 | 2022 |
| 2013 | 2018 | 2023 |
| 2014 | 2019 | 2024 |

- 12.15 **If so, how?**

How are results aggregated, presented, how often reported and how are interpreted; epidemiologically or statistically?

- 12.16 **Is there a long-term strategy for Chemicals and health-related biomarkers WES in your country?**

Yes No

- 12.17 **Is there a data ownership policy?**

Yes No

- 12.18 **Who owns the raw and processed data?**

Water utilities own the data

Public health institutions own the data

Laboratory/Service providers own the data Open data

Municipalities own the data University or research center I don't know

Other

- 1. **Other or additional relevant information about data ownership.**
  2. **Rate the main challenges for the Chemicals and health-related biomarkers WES system in your country or the areas where support would be most needed.**
  - **Not challenging**

****Slightly Challenging**

*****Moderately Challenging**

******Very Challenging**

*******Extremely Challenging**

| **Coordination**: lack of coordination among Ministries or other leading organizations |  |
| --- | --- |
| **Funding**: lack of financial support to ensure operationalization and sustainability |  |
|  |  |

| **Sampling**: challenges with the logistics to secure the collection of samples and consequent analysis |  |
| --- | --- |
| **Operationalization**: consistency in testing frequency and analysis between varying sites and laboratories running the samples |  |
| **Analysis**: verification of methods due to lack of laboratory capacities and/or experience. QA  /QC, reference materials/inter-labs |  |
| **Estimates**: difficulties with data normalization, modelling and interpretation |  |
| **Data warehouse**: no centralized IT system for wastewater surveillance data storage, access or sharing |  |
| **Integration**: translation and integration of the final results into clinical surveillance and response systems |  |

# Additional questions

- 13.1 **How was the information of this survey gathered?**

Individual experts responded

A group of experts discussed and provided a shared understanding Other

13.2 **Other**

- 13.3 **Did you organize a national workshop to gather the responses?**

Yes, online Yes, hybrid Yes, on-site No

- 1. **If you organized a workshop, please provide a word file with: number of participants, date(s), program and list of participants**

## Statistics

- 1. **For which audience do you provide statistical results of wastewater surveillance in your country?**

general public

internal for authorities/decision makers expert groups

no statistical analysis provided

- 1. **Do you have success stories regarding the provision of statistical results in wastewater surveillance in your country?**
  2. **In which areas do you experience a need for (more) statistical analysis for wastewater surveillance?**
  3. **What are the main barriers to the use of statistical analysis for wastewater surveillance data?**
  4. **Which problems related to the communication of statistical results do/did you encounter in your country?**
  5. **How do you ensure that shared results are understandable for different target audiences?**
  6. **Do you have examples of communication statistical results for different audiences?**
