# Supplementary material for: Towards the institutionalization of wastewater surveillance for public health: results from the EU-WISH mapping survey
Source: Eur J Public Health. 2026 Jan 14;36(2):ckaf259. doi: 10.1093/eurpub/ckaf259 (PMC13017784; doi:10.1093/eurpub/ckaf259)
Supplement: ckaf259_Supplementary_Data [file ckaf259_supplementary_data.zip › ejph-2025-07-om-0562-File007.docx]

**Table S2**. Consultation processes used by participating countries to prepare their national responses to the EU-WISH system-mapping survey. The table indicates whether each country organised a national workshop or multi-stakeholder consultation (on-site, online, or hybrid), or whether inputs were gathered through alternative mechanisms such as email exchanges, internal expert discussions, or smaller meetings (answering “no”).

| **Country** | **Did you organize a national workshop to gather the responses?** |
| --- | --- |
| Latvia | No |
| Croatia | Yes, on-site |
| Estonia | Yes, online |
| Netherlands | No |
| Greece | No |
| Ireland | Yes, online |
| Slovenia | No |
| France | No |
| Austria | No |
| Italy | Yes, online |
| Ukraine | No |
| Norway | No |
| Portugal | No |
| Luxembourg | Yes, on-site |
| Germany | No |
| Spain | No |
| Lithuania | No |
| Finland | Yes, online |
| Denmark | Yes, online; Yes, on-site |
| Hungary | No |
| Romania | No |
| Cyprus | Yes, online |
| Sweden | No |
| Slovak Republic | No |
| Czechia | Yes, hybrid |
| Belgium | No |
| Malta | No |
